# Identifications of QTLs and Candidate Genes Associated with *Pseudomonas syringae* Responses in Cultivated Soybean (*Glycine max*) and Wild Soybean (*Glycine soja*)

**DOI:** 10.3390/ijms24054618

**Published:** 2023-02-27

**Authors:** Jinhui Wang, Haojie Feng, Xiaoke Jia, Shengnan Ma, Chao Ma, Yue Wang, Siyang Pan, Qingshan Chen, Dawei Xin, Chunyan Liu

**Affiliations:** 1 Key Laboratory of Soybean Biology in Chinese Ministry of Education, College of Agriculture, Northeast Agricultural University, Harbin 150030, China; 2 Agricultural Technology Extension Station of Heilongjiang Province, Harbin 150036, China

**Keywords:** soybean, bacterial spot disease, *Pseudomonas syringae*, wild soybean, QTL

## Abstract

Soybeans (*Glycine max*) are a key food crop, serving as a valuable source of both oil and plant-derived protein. *Pseudomonas syringae pv. glycinea* (*Psg*) is among the most aggressive and prevalent pathogens affecting soybean production, causing a form of bacterial spot disease that impacts soybean leaves and thereby reduces crop yields. In this study, 310 natural soybean varieties were screened for *Psg* resistance and susceptibility. The identified susceptible and resistant varieties were then used for linkage mapping, BSA-seq, and whole genome sequencing (WGS) analyses aimed at identifying key QTLs associated with *Psg* responses. Candidate *Psg*-related genes were further confirmed through WGS and qPCR analyses. Candidate gene haplotype analyses were used to explore the associations between haplotypes and soybean *Psg* resistance. In addition, landrace and wild soybean plants were found to exhibit a higher degree of *Psg* resistance as compared to cultivated soybean varieties. In total, 10 QTLs were identified using chromosome segment substitution lines derived from Suinong14 (cultivated soybean) and ZYD00006 (wild soybean). *Glyma.10g230200* was found to be induced in response to *Psg*, with the *Glyma.10g230200* haplotype corresponding to soybean disease resistance. The QTLs identified herein can be leveraged to guide the marker-assisted breeding of soybean cultivars that exhibit partial resistance to *Psg*. Moreover, further functional and molecular studies of *Glyma.10g230200* have the potential to offer insight into the mechanistic basis for soybean *Psg* resistance.

## 1. Introduction

Soybeans (*Glycine max*) are an important agricultural crop, serving as an important source of oil and high-quality plant protein [1,2,3]. In China, Heilongjiang is the site of over 40% of domestic soybean production [4,5]. The yield and quality of soybean crops have been seriously impacted by a series of diseases in recent years, with bacterial spot disease (BSD) caused by *Pseudomonas syringae pv. glycinea* (*Psg*) being one important cause of soybean yield losses [6,7,8]. *Psg* can overwinter and infect soybean plants even under low-temperature and high-humidity conditions [9]. While it can infect the pods, stems, and petioles of soybean plants, this pathogen primarily causes damage by infecting the leaves [6]. During the early stages of infection, polygonal, transparent, water-stained spots develop, with light yellow spots developing as the disease progresses [7]. These spots are often brown in the middle and are surrounded by a chlorotic halo, with affected leaves readily falling off of infected plants [10,11,12]. BSD outbreaks are primarily observed in Northeast China and in the Huang-Huai-Hai region, resulting in yield losses for soybeans grown under both field conditions and in greenhouses [4,5,13].

Efforts to breed *Psg*-resistant soybeans present a sustainable and effective approach to reducing BSD-related damage to soybean plants [14]. *Psg* resistance is a complex, quantitative trait under the control of multiple genes, and as such, marker-assisted selection can be used to help breed resistant cultivars via the integration of the known resistance locus [14,15,16,17]. However, resistance-related loci and genes identified to date are highly diverse and are dispersed among different germplasms, complicating efforts to develop *Psg*-resistant soybean varieties [14]. There is thus a clear need to further characterized *Psg* resistance and associated QTLs and genes in available soybean germplasm resources so as to guide future breeding and genome editing. Multiple *Psg* resistance-related genes have been identified to date. For example, Rpg-1 is a CC-NB-LRR protein that can aid in plant resistance to *Psg* carrying the type III effector AvrB [18,19,20], while *Rpg-2* is related to soybean resistance to *Psg* carrying the effector AvrA [20]. The *GmEDS1a/b* and *GmPAD4* genes are required for *Rpg2*-mediated resistance to *Psg*, with the GmEDS1 protein being capable of directly interacting with pathogen-derived AvrA1 to control its function in *Psg*-infected soybean plants [21,22,23]. *Rpg3* and *Rpg4* are additionally associated with resistance against *Psg* expressing AvrB2 and AvrD1, respectively [24]. GmRIN4b can be phosphorylated by AvrB, affecting direct interactions between Rpg1 and GmRIN4b and thereby contributing to *Psg* resistance [25]. The resistance-related protein GmNDR1 can additionally directly interact with GmRIN4 [25]. While these studies clearly demonstrate that certain *Psg* resistance-related genes have been functionally characterized to date, these genes primarily focus on effector-related signaling pathways, and there have been few comprehensive analyses of soybean *Psg*-resistance genes to date [7].

QTL mapping can be used as an effective means of identifying key loci associated with particular agronomic traits and aiding in molecular marker-assisted selective breeding [26,27,28]. While there have been few *Psg*-related QTL studies on soybean to date, there have been several such studies in other crop species. For example, four QTLs associated with resistance to *Pseudomonas syringe pv. tomato* (*Pst*) have been identified on three chromosomes via a composite interval mapping approach [29]. High-density genetic maps and intensive phenotyping have further led to the identification of seven QTLs in kiwifruit, with the candidate genes within these QTLs having been further validated via RNA sequencing as being related to *Pseudomonas syringae pv. Actinidiae* (*Psa*) resistance [15]. In total, 76 QTLs related to *Pseudomonas syringae pv. Phaseolicola* (*Psp*) resistance have been identified in common beans, with 26 of these QTLs being positive for resistance-associated gene clusters encoding nucleotide-binding and leucine-rich repeat proteins and some known defense genes [30]. QTL-based mapping of BSD-resistant crops thus represents an effective means of characterizing resistance mechanisms, highlighting the value of employing a comparable approach to characterize QTLs associated with soybean *Psg* resistance.

The present study was developed to identify BSD-related QTLs and genes. To that end, the BSD-susceptible cultivated variety Suinong14 and the BSD-resistant wild soybean variety ZYD00006 were used as parents to construct a population of chromosome segment substitution lines (CSSLs). These CSSLs were then used to identify QTLs associated with soybean *Psg* responses through a combination of QTL mapping, BSA-seq, and analyses of the insertion of wild soybean chromosomes. Candidate genes within the QTL interval were further screened and validated through qPCR and haplotype analyses.

## 2. Results

### 2.1. Suinong14 (Improved Cultivar) and ZYD00006 (Wild Soybean) Exhibit Distinct Phenotypic Responses to Psgneau001

**Figure 1:**
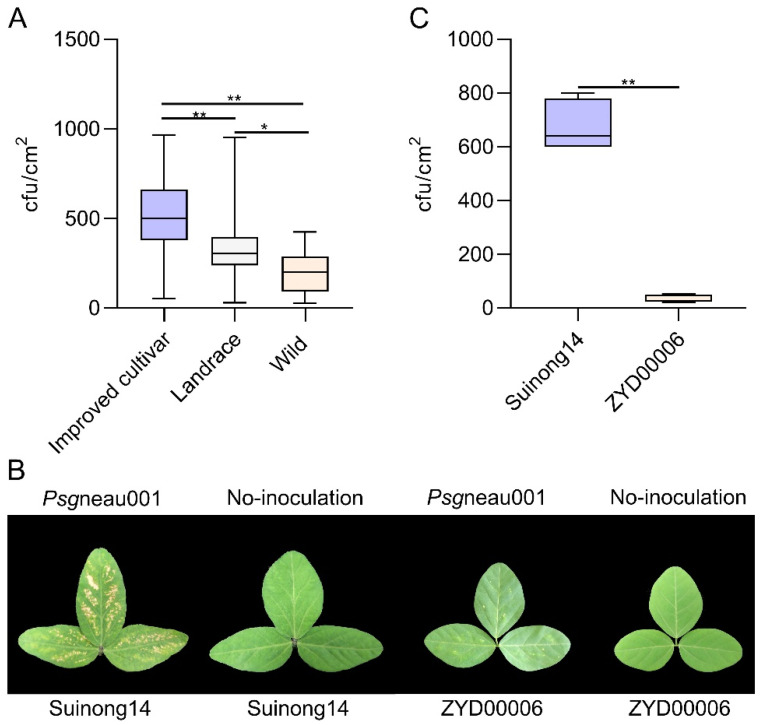
Phenotypic analysis of soybean varieties’ responses to *Psg* under greenhouse conditions. (**A**) *Psg* resistance phenotypes for 310 soybean varieties, including 229 improved cultivars, 71 landraces, and 10 wild soybean varieties. (**B**) The phenotypes of Suinong14 (susceptible variety) and ZYD00006 (resistant variety) following *Psg* inoculation. (**C**) Phenotypes of Suinong14 and ZYD00006 leaves following infection with or without *Psg*. Asterisks denote Student’s *t*-test significance (* indicates *p* < 0.05, ** indicates *p* < 0.01).

A total of 310 soybean germplasms from Northeast China, including 229 improved cultivars, 71 landraces, and 10 wild soybean varieties, were collected to evaluate the resistance of different soybean varieties’ responses to *Psg*neau001 (Table S1). Phenotypic analyses of the 310 soybean germplasms revealed that the wild accessions exhibited pronounced BSD resistance and were less affected by BSD as compared to the tested landraces and improved cultivars. In contrast, improved soybean cultivar accessions exhibited the highest degree of pathogenicity, with many exhibiting clear symptoms of disease, while tested landraces exhibited intermediate disease phenotypes between those of wild soybeans and improved cultivars (Figure 1A). One possible explanation for this observed resistance pattern may be that *Psg* has gradually developed the ability to overcome resistance in cultivated soybean plants through the natural variation and selection of industrial bactericide. Alternatively, artificial soybean domestication and agronomic trait improvement may have reduced the genetic diversity present among cultivated soybean varieties, leading to reduced *Psg*neau001 resistance owing to an overall narrowing of the genetic background. Importantly, these results suggest that wild soybean accessions represent a valuable source of abundant *Psg* resistance-related loci. Through an analysis of the resistance and susceptibility to *Psg*, the wild soybean could be used to improve the resistance of soybean cultivars to *Psg*, especially for the improvement of soybean varieties with relatively large spread areas. ZYD00006, one of the 10 wild soybean varieties included, was highly resistant to *Psg* (Figure 1B,C). Suinong14, as one of the 229 improved cultivars, is a variety with a large area of popularization in Northeast China, and a large number of new soybean varieties have come from Suinong14. While Suinong14 was highly susceptible to *Psg*neau001 (Figure 1B,C), it had great influence on yield and quality. In order to identify important QTLs and genes affecting resistance to *Psg* for improving the improved cultivar, a population of one hundred and seventy chromosome segment substitution lines (CSSLs) derived from cross and continuous backcross between Suinong14 (recurrent parent) and ZYD00006 (donor parent) were constructed to identify QTL loci response to *Psg*neau001. From 2006, when all 170 CSSLs were inoculated with *Psg*neau001, the population exhibited significant variability with respect to resistance, with CFU (colony forming unit) counts ranging from 60 to 1102 across the 170 CSSLs. The phenotypes of the parent were located within these intervals (Table 1). This suggests that the genetic differences between Suinong14 and ZYD00006 may contribute to the observed differences in response to *Psg*neau001.

### 2.2. Identified 10 QTLs for BSD in Soybean CSSL Populations

In total, 10 QTLs associated with disease resistance were detected using a WinQTL Cartographer via a composite interval mapping model (CIM) [27,28,29], including *qbsd-02-1*, *qbsd-08-1*, *qbsd-08-2*, *qbsd-08-3*, *qbsd-09-1*, *qbsd-10-1*, *qbsd-10-2*, *qbsd-11-1*, *qbsd-11-2*, and *qbsd-16-1* located on chromosomes 2, 8, 9, 10, 11, and 16 (Table 2).

### 2.3. 3 Intervals Associated with Psgneau001 Resistance Identified via BSA-seq

**Figure 2:**
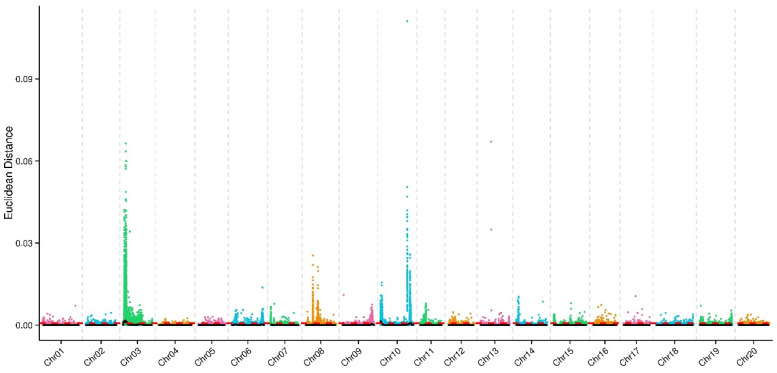
Euclidean distance (ED)-associated value distributions on soybean chromosomes. Note: chromosome names are denoted on the *X*-axis, while colored dots correspond to the ED value for each SNP locus. The black line is the fitted ED value, while the red dotted line corresponds to the associated significance threshold, the candidate interval associated with the trait was determined by using 0.0007 (median plus three standard deviations) as a standard threshold for CFU.

By comparing the sequencing data of 30 resistant varieties and 30 susceptible varieties of CSSLs, Suinong14, and ZYD00006 to the reference genome, a total of 3,739,321 SNPs of response to *Psg*neau001 were used for correlation analysis by the Euclidean distance method. According to the rules of this method, we excluded SNP loci containing multiple alleles (those with more than one SNP within 5 bp) and SNP loci with identical genotypes between the two pools. Finally, we obtained 3,099,344 high-quality SNPs, including 630,809 mixed-pool genotype-consistent loci and 9168 multiple-allele loci. An SNP index association algorithm was then used for candidate gene selection, with the Euclidean distance method being used to fit the ΔSNP index. Three intervals correlated with disease resistance phenotypes were thereby located based on the results of a computer simulation test including Chr03 (chromosome location: 0.67–15.37 cM, physical location: 0.38–7.39 Mb), Chr10 (chromosome location: 0–5.90 cM, physical location: 0–3.15 Mb), and Chr10 (chromosome location: 84.26–92.52 cM, physical location: 43.91–48.05 Mb) (Figure 2) (Table 3).

### 2.4. Candidate QTL Identification

By comparing overlapping QTL intervals from QTL mapping and BSA-seq, a candidate QTL extending from 45.3 Mb to 48.2 Mb on Chromosome 10 was identified and selected for further analyses of BSD resistance-related genes.

### 2.5. Chromosome Fragment Insertion-Based Candidate Interval Verification

**Figure 3:**
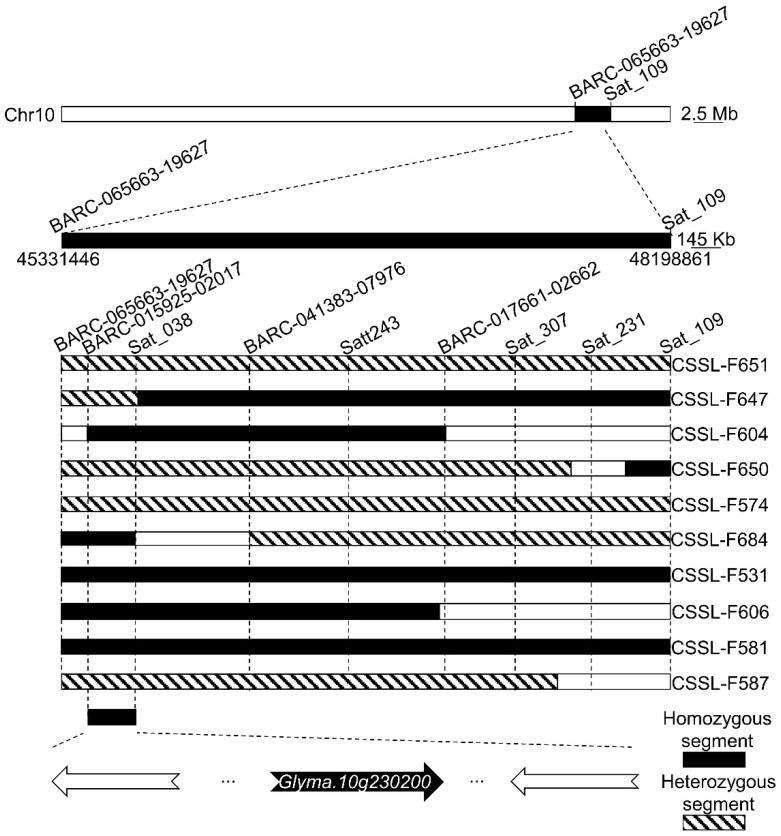
Genetic linkage analysis-based fine mapping of segregants exhibiting divergent phenotypes induced in response to *Psg*. The black and striped sections represent the chromosome fragments of ZYD00006, and the black sections indicate the homozygous fragments, while the striped sections indicate the heterozygous fragments. The distributions of substituted wild soybean chromosomal segments in these CSSLs were used to narrow the location of the *Psg*-related gene(s) to a 301.9 kb region between the markers 015925-02017 and Sat_038.

Over the multiple years of backcrossing, DNA fragments from ZYD00006 were integrated into the Suinong14 genome such that the genomes of individual members of the constructed CSSLs primarily consisted of the Suinong14 genome containing small segments of the ZYD00006 genome. Because of the difference in *Psg*-resistance between Suinong14 and ZYD00006, the genomic integration led to different CSSLs exhibiting a distinct genomic composition as compared to that of Suinong14, resulting in distinct *Psg*-resistance phenotypes in these individual CSSLs. Via the screening of 10 extremely resistant individuals from these CSSLs, certain ZYD00006 DNA fragments were inserted into the region of Chromosome 10 that overlapped with the candidate QTL identified above. Based on analyses of the overlapping inserted DNA fragments and BSD susceptibility phenotypes, a 301.9-kb locus (45.8–46.1 Mb) between the BARC-015925-02017 and Sat_038 markers was identified as potentially being associated with *Psg*neau001 infection (Figure 3), indicating that one or more genes within this region may interact with *Psg*neau001 to control associated disease resistance.

### 2.6. Glyma.10g230200 Identified as the Candidate Gene Response to Psg via Sequencing and Expression Analysis

**Figure 4:**
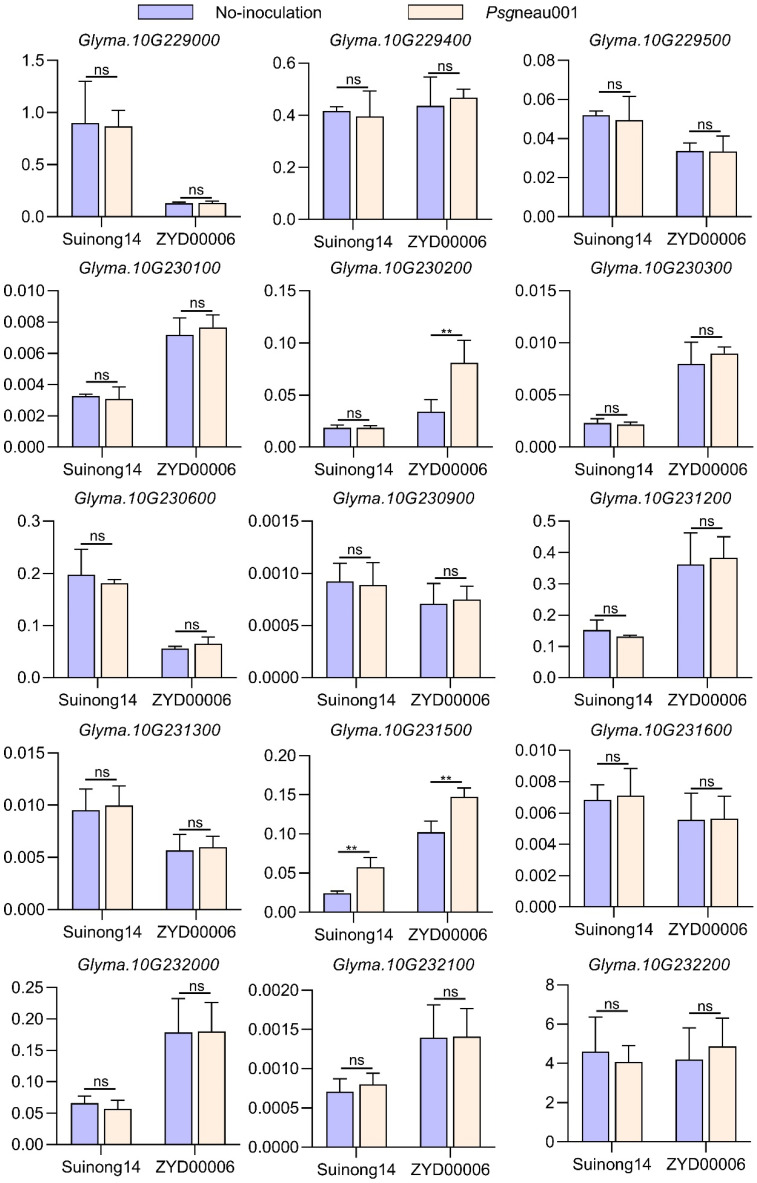
Analysis of relative candidate gene expression within the QTL interval as assessed via qPCR. Relative gene expression was assessed via the 2^−ΔΔCt^ method [4,14,28], with *GmUKN1* (*Glyma.12g020500*) being used for normalization. Data are presented as means ± SE from three replicates. Asterisks denote Student’s *t*-test significance (** indicates *p* < 0.01 and ns indicates no significance) of data.

Based on the Williams82 reference genome, 40 genes were located within the 301.9-kb genomic region of the candidate QTL. To explore which of these genes were associated with *Psg*neau001 resistance, SNP distributions for this 301.9-kb region of Suinong14 and ZYD00006 were assessed via whole-genome resequencing, revealing 1279 SNPs within this region of Chromosome 10 (Figure S1). Of these SNPs, 127 were located within the exonic regions of 30 genes, while 88 were located within the 3000-bp promoter regions upstream of 20 genes, including 20 shared genes (Table S2). A qPCR analysis was next used to assess the expression of these 30 genes potentially associated with *Psg*neau001 resistance in Suinong14 and ZYD00006 (Table S3). Of these 30 genes, 15 (*Glyma.10G228500*, *228600*, *228800*, *228900*, *229200*, *229300*, *229600*, *229800*, *230000*, *230400*, *230700*, *230800*, *231000*, *231100*, and *231400*) were not expressed at detectable levels. The expression patterns of 13 of the 15 remaining genes did not differ significantly when comparing with plants that were not inoculated with *Psg*neau001 (Figure 4). The *Glyma.10g231500 Psg*neau001 infection could increase its expression level both in Suinong14 and ZYD00006. Though *Glyma.10g231500* might be involved in response to *Psg*neau001, we could not select it as the candidate gene, because this expression pattern was similar in Suinong14 and ZYD00006. However, *Glyma.10g230200* was upregulated almost two-fold following *Psg*neau001 inoculation in ZYD00006, whereas its expression did not change following inoculation in Suinong14 (Figure 4). According to the SNP analysis of the *Glyma.10g230200* in parents, there were two SNPs in the exons and 15 SNPs in the promoter region, and the SNPs in exons do not cause a change in amino acids (Table S2). It indicated that the SNPs in the promoter region could cause different expression patterns of *Glyma.10g230200* during *Psg*neau001 infection. Thus, we selected *Glyma.10g230200* as the candidate gene response to *Psg*neau001 in soybean.

### 2.7. Glyma.10g230200 Encodes a WRKY27 and a Gene Differentially Expressed in BSD-Resistant and -Susceptible Soybeans Inoculated with Psgneau001

**Figure 5:**
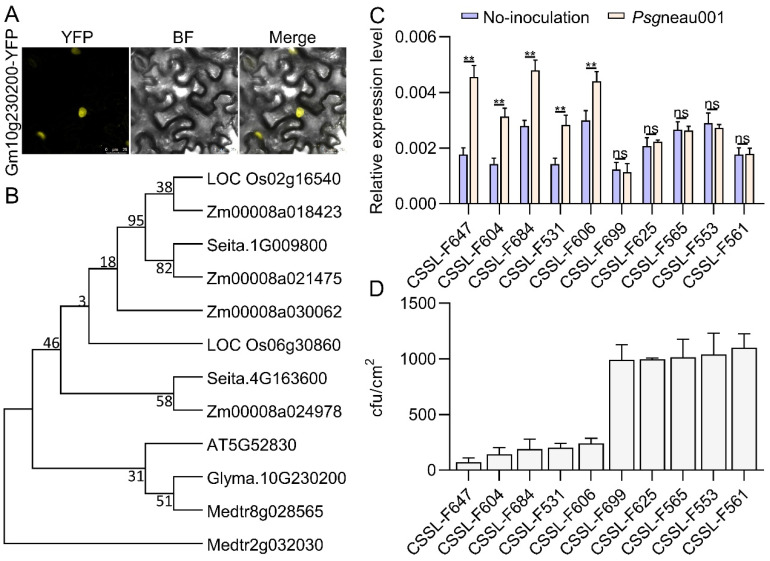
Information about *Glyma.10g230200* and the relative expression of in different susceptible and resistant CSSL accessions. (**A**) Analysis of subcellular localization of *Glyma.10g230200* fused to YFP (yellow fluorescent protein), where BF indicates bright field; bars = 25 μm. (**B**) Maximum likelihood phylogenetic analysis of the *Glyma.10g230200* tree of different plants (including *Glycine ma*x, *Oryza sativa*, *Zea mays*, *Setaria italica*, *Arabidopsis thaliana,* and *Medicago truncatula*) created with MEGA7. (**C**) *Glyma.10g230200* gene expression in five extremely susceptible CSSLs and five extremely resistant CSSLs following *Psg*neau001 inoculation (24 hpi). Data are presented as means ± SE from three replicates. Asterisks denote Student’s *t*-test significance (** indicates *p* < 0.01 and ns indicates no significance) of data. (**D**) Phenotypes of Hap1 and Hap2 following *Psg*neau001 inoculation in five *Psg*-resistant germplasms (CSSL-F647, CSSL-F604, CSSL-F684, CSSL-F531, and CSSL-F606) and five *Psg*-susceptible germplasms (CSSL-F699, CSSL-F625, CSSL-F565, CSSL-553, and CSSL-F561).

The 894-bp *Glyma.10g230200* gene encodes a 297 amino acid WRKY family protein and targets the plant nucleus (Figure 5A). Phylogenetic analyses of six plants (including *Glycine max*, *Oryza sativa*, *Zea mays*, *Setaria italica*, *Arabidopsis thaliana,* and *Medicago truncatula*) revealed *Glyma.10g230200* to be closely related to *Medtr8g028565* and *AT5G52830*(Figure 5B). Via phenotypic analysis, the incidence of leaf disease was investigated at Grade 0 to 5: Grade 0: leaf lesions are more difficult to see, and CFU is 0–100; Grade 1: leaf lesions are not obvious, CFU is 100–200; Grade 2: the lesion area is enlarged with a chlorosis halo around it, CFU is 200–400; Grade 3: the lesion area has spread, CFU is 400–600; Grade 4: lesion area encompasses more than half of the leaf area, leaf is yellow, CFU is 600–800; Grade 5: leaves turn yellow and then wilt or fall, CFU exceeds 800. Grades 0 and 1 obviously indicate disease resistance, and Grades 4 and 5 obviously indicate susceptibility. According to the CFU and the grade of leaf disease, the resistant and sensitive cultivar were evaluated. Ten representative CSSLs, including five that were extremely resistant to BSD (CSSL-F647, CSSL-F604, CSSL-F684, CSSL-F531, and CSSL-F606) and five that were extremely susceptible (CSSL-F699, CSSL-F625, CSSL-F565, CSSL-553, and CSSL-F561) were used to analyze *Glyma.10g230200* expression at 24 h post *Psg*neau001 inoculation. There were no significant differences in *Glyma.10g230200* expression in response to *Psg* inoculation in the five extremely susceptible CSSLs, whereas it was significantly upregulated at 24 h post inoculation in the five extremely resistant CSSLs (Figure 5C,D) This confirmed that *Glyma.10g230200* could be involved in resistance to *Psg*neau001.

### 2.8. Haplotype Analyses Suggest a Link between Glyma.10g230200 and Psgneau001 Resistance

**Figure 6:**
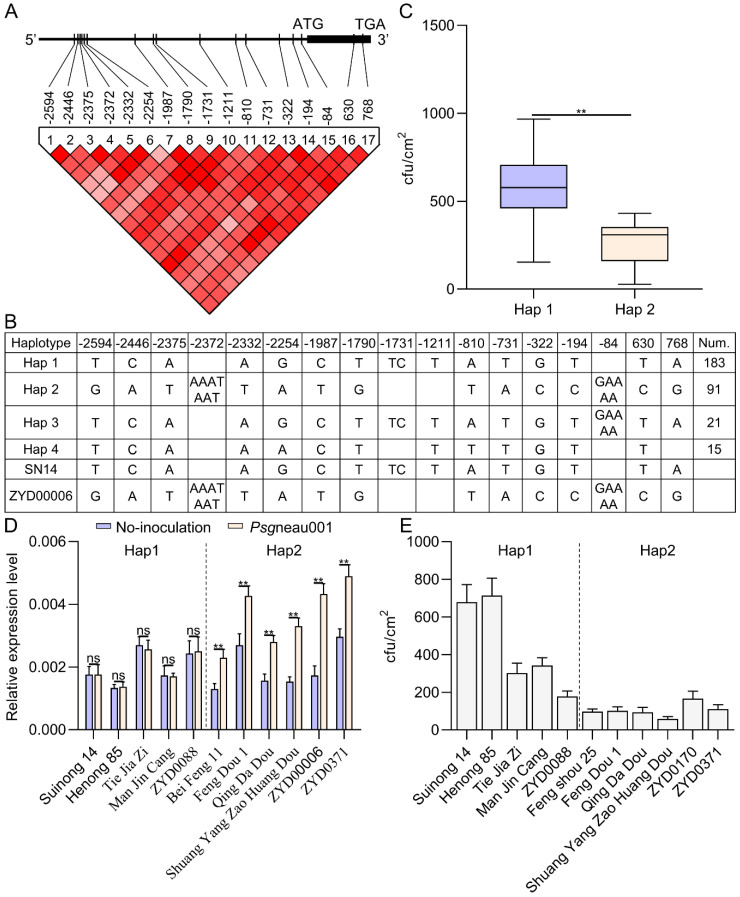
Haplotype analysis of *Glyma.10g230200*. (**A**,**B**) Haplotype analysis of *Glyma.10g230200* in 310 soybean accessions. (**C**) Phenotypes of Hap1 and Hap2 following *Psg*neau001 inoculation. (**D**) *Glyma.10g230200* gene expression in some Hap1 and Hap2 soybean varieties following *Psg*neau001 inoculation (24 hpi). Data are presented as means ± SE from three replicates. Asterisks denote Student’s *t*-test significance (** indicates *p* < 0.01 and ns indicates no significance) of data. (**E**) Phenotypes of Hap1 and Hap2 following *Psg*neau001 inoculation.

Given that different soybean germplasms exhibited distinct phenotypic responses to *Psg*neau001, in an effort to confirm whether resistance to this pathogen is associated with the *Glyma.10g230200* gene, the resequencing and *Psg*neau001 resistance of the 310 soybean natural varieties used in this study were assessed to analyze *Glyma.10g230200* haplotypes. In total, four *Glyma.10g230200* haplotypes (Haps) were identified in these 310 soybean varieties, with the two Haps containing over 10 accessions being considered to be dominant. Only one SNP and one indel were identified within the promoter regions for Hap 1 (including Suinong14) and Hap 2 (including ZYD00006) (Figure 6A,B). To explore the relationship between different *Glyma.10g230200* alleles and variations in resistance to *Psg*neau001, a combined phenotype and haplotype analysis was conducted using these 310 soybean germplasms. This analysis revealed a marked difference in *Psg*neau001 resistance between Hap1 and Hap2 soybean accessions (Figure 6C). Eleven natural soybean varieties, including five extremely susceptible varieties (Hap1) and six extremely resistant varieties (Hap2), were randomly selected to analyze *Glyma.10g230200* expression patterns at 24 h post *Psg*neau001 inoculation. As shown in Figure 6D,E, *Glyma.10g230200* expression in five extremely susceptible varieties was not significantly altered following *Psg*neau001 inoculation, whereas it was significantly upregulated at 24 h post inoculation in six of the tested extremely resistant varieties. This haplotype analysis thus confirmed the relationship between *Glyma.10g230200* and soybean resistance to *Psg*neau001 infection.

## 3. Discussion

Soybean is a critically important crop for agricultural production, with stable soybean yields being dependent on the ability to prevent disease. BSD caused by *Pseudomonas syringae pv. glycinea* (*Psg*) can have a serious adverse impact on soybean quality and crop yield. Certain genes and loci associated with *Psg* resistance have been identified in previous genetic and RNA-seq studies, including *Rpg-1* [24], *Rpg-2* [24], *GmEDS1a/b* [21], *GmPAD4* [22,23], *GmRIN4b* [25], and *GmNDR1* [24]. Identifying additional functional genes and loci associated with *Psg* resistance has the potential to offer new insight into the molecular basis for breeding improved soybean varieties that are less susceptible to BSD.

Different soybean germplasms are highly genetically diverse and have the potential to aid in soybean breeding and analyses of key agronomic traits [33,34,35]. These germplasms are broadly divided into improved cultivars, landraces, and wild soybeans. Improved cultivars were originally derived from wild soybeans, with their genetic diversity having been gradually reduced over the course of domestication and selection for desirable agronomic traits [3,28,36,37,38]. Relative to improved cultivars, wild soybeans exhibit a higher degree of genotypic and phenotypic diversity [39,40,41]. When not subject to artificial selection, wild soybeans can adapt to a range of environmental conditions and abiotic stressors through natural selection, in addition to acquiring biotic stress resistance [4,39,42,43]. In this study, 10 wild soybeans, 71 landraces, and 229 improved cultivars were leveraged to identify QTLs and genes associated with *Psg* resistance in these different germplasm resources. Consistent with their greater genetic diversity, the tested wild accessions exhibited greater *Psg* resistance compared with improved cultivars, suggesting that they represent a valuable resource for efforts to breed resistant soybean varieties. The breeding of the Suinong14 (improved cultivar) and ZYD00006 (wild soybean) varieties was then conducted as a means of further probing *Psg* resistance-related QTLs and genes. CSSLs were generated via extensive backcrossing onto the Suinong14 background, with molecular markers and resequencing then being used to determine the location of ZYD00006 chromosome fragment insertion [44,45,46]. As such, backcrossing was continuously performed for over 10 years, the ZYD00006 DNA fragments were inserted into the Suinong14 genome, and different individuals exhibited distinct genomic compositions capable of reducing the interfering effect of the genetic background on these analyses [47,48,49,50]. Suinong14, an improved cultivar, exhibits greatly reduced genetic diversity and increased linkage disequilibrium relative to that observed for wild soybean varieties as a consequence of domestication, and these properties are poorly suited to the mapping of QTLs associated with key agronomic traits [48]. The ZYD00006 variety is found in the wild in China and was the wild variety most closely related to the improved cultivars [51]. Because of its more allelic diversity compared with cultivated soybeans [34,39,51,52], wild soybean can be used as an ideal germplasm to detect loci associated with particular traits and improve important agronomic traits, by the combination of wild and cultivated soybeans [34,43]. Here, 10 QTLs associated with *Psg* resistance were identified, with some of these exhibiting overlap with previously reported disease resistance-related genes or loci, including *qXav-10* [4], *q10.1* [31], *qSCN3-11* [32], and *qXav-16* [4], respectively. These data thus supported the overall accuracy of these QTL mapping and genetic analysis efforts. Bulked segregant analysis (BSA) and chromosome fragment insertion analysis approaches were then further used to screen for and analyze *Psg* resistance loci in an effort to further refine these results.

Through gene annotation, parents’ genome information, and qPCR analyses of the overlapping QTLs, *Glyma.10g230200* was found to be related to *Psg*neau001. *Glyma.10g230200* encodes a 297 amino-acid WRKY family protein detectable in the roots and leaves of soybean plants. WRKY proteins comprise a large family of plant transcription factors that contain one or two conserved ~60 amino acid WRKY domains that bind to W-box elements [TTGAC(C/T)]. These WRKY domains contain a conserved WRKYGQK sequence followed by C2H2 or C2HC zinc-finger motifs [53]. A growing number of studies have shown WRKY transcription factors to serve as important negative or positive regulators of plant response to specific abiotic and biotic stress [54,55]. For example, several *WRKY* genes are rapidly induced in response to pathogen exposure, leading to the activation of stress-related gene expression that can defend plants against inducing stressors or promote stress tolerance [56]. The most recent research suggests that WRKY proteins can additionally interact with other transcription factors through complex signaling networks that can lead to the regulation of a diverse range of defense-related genes and processes to coordinate plant immunity [54,55]. For example, the OsWRKY62 protein can negatively regulate both effector-triggered immunity (ETI) and PAMP (pathogen-associated molecular patterns)-triggered immunity (PTI), inhibiting the expression of a subset of genes associated with defensive responses [57]. In the context of *Xanthomonas oryzae pv. Oryzae* infection, OsWRKY62 can reportedly positively regulate ETI and PTI [58,59]. *AtWRKY27* expression in *Arabidopsis thaliana* can reportedly suppress defensive responses against *Ralstonia solanacearum* by modulating gene expression. Moreover, the CaWRKY40b protein expressed in pepper can control immunity-associated gene expression in the context of *Ralstonia solanacearum* infection [60]. The virus-induced gene silencing (VIGS) of *CaWRKY40b* or *CaWRKY40b-SRDX* (the chimeric repressor version of *CaWRKY40b*) overexpression respectively resulted in the up- and down-regulation of positive and negative regulatory genes, while the opposite effect was observed following the overexpression of *CaWRKY40b* [60]. WRKY family proteins can thus play disparate roles in response to biotic stress exposure [54,55]. In total, 174 *WRKY* genes have been identified in the soybean genome, several of which have been linked to biotic stress responses [53,61]. For example, GmWRKY31 and GmHDL56 interact to regulate soybean defense-associated gene expression so as to resist *Phytophthora sojae* [62]. Similarly, *GmWRKY40* positively regulates defensive responses against *P. sojae* by promoting JA signaling and H_2_O_2_ accumulation [63]. Previously, 22 different soybean *WRKY* genes were found to be differentially expressed in response to *Peronospora manshurica*, with GmWRKY31 reportedly binding the *GmSAGT1* promoter to drive its upregulation and thereby enhance resistance against this pathogen [56]. Here, *Glyma.10g230200* was identified as a regulator of soybean response to *Psg*neau001 in both cultivated and wild soybean plants. *Psg*neau001 can induce *Glyma.10g230200* expression in disease-resistant soybean cultivars, and SNPs in the promoter region of this gene were associated with differences in its expression patterns among soybean varieties. Further analyses of the phenotypes and *Glyma.10g230200* haplotypes of natural soybean varieties revealed a significant correlation between the *Glyma.10g230200* haplotype and BSD resistance. Through the whole genome resequencing of natural varieties and analysis of expression levels in Suinong14 and ZYD00006, we confirmed that the SNPs in the promoter region are closely associated with phenotypic differences between Suinong14 and ZYD00006 in response to *Psg*neau001. Though an analysis of cis-acting elements of different promoter sequences in Hap 1 and Hap 2, the *Glyma.10g230200* promoter of ZYD00006 lacked one GC motif and some unnamed motives compared with Suinong14 (Tables S4 and S5). GC motifs have been found to be involved in stress and hormone response [64,65,66]. The difference between the GC motif and other motifs in the promoter of Suinong14 and ZYD00006 might result in a different expression pattern response to *Psg*neau001, leading to the differences in resistance to *Psg*neau001. These results thus support the notion that *Glyma.10g230200* is a regulator of *Psg*neau001 resistance and provide a foundation for further studies on the mechanistic basis for such resistance. These findings will also support efforts to breed BSD-resistant soybean varieties to improve crop yield and quality.

## 4. Materials and Methods

### 4.1. Bacterial Strain Selection

The experimental *Pseudomonas* strain used in this study, *Psg*neau001, was isolated from soybean leaves affected by BSD in the main soybean planting area of Jiamusi city, Heilongjiang province, China. *Psg*neau001 was isolated as follows [4]. Soybean leaves with bacterial spot disease were collected from soybean plants grown in the main producing areas of Jiamusi city after rain. The collected leaves were sterilized with 1:1000 HgCl_2_, after which they were immersed in alcohol and subsequently thoroughly rinsed with sterilized water to remove the HgCl_2_. The leaves were transferred to sterilized tubes and ground using a sterilized pestle, and after a brief centrifugation, the supernatant was coated on the NYG solid medium. Candidate single clones were underlined on new NYG solid medium, and then the new single clones were picked for subsequent verification to ensure the purity of the bacteria. New candidate single clones were isolated. More than 2 days were required for this clone to grow at 28 °C, after which the *16S rDNA* from single clones were amplified and sequenced (primer sequence as shown in Table S3). The BLAST analysis of the NCBI database (https://blast.ncbi.nlm.nih.gov/, accessed on 12 December 2021) with the *16Sr DNA* of candidate clones were used to determine *Pseudomonas*. The candidate *Pseudomonas* strain suspension was prepared by diluting cultured *Psg* 1000-fold using 10 mM sterilized MgCl_2_ and 0.05% Silwet-L77 when the bacteria had reached an OD_600_ (optical density) of 0.8 when cultured at 28 °C. Suspended bacteria were applied to the back of trifoliate in Suinong14 using an atomizer, after which plants were housed in a humidified cabinet at 100% relative humidity for 4 days. The strain could cause significant symptoms of BSD; thus, the isolated strain was named *Psg*neau001.

### 4.2. Plant Materials

In total, 310 natural soybean varieties were used for disease susceptibility analyses. These soybean germplasm resources consisted of 10 wild soybeans (*G. soja* Sieb. & Zucc.), 71 landraces, and 229 improved cultivars. BSD-related QTLs were verified using 170 chromosome segment substitution lines (CSSLs) derived from the crossing and continuous backcrossing of the soybean cultivar Suinong14 (which showed obvious susceptibility to *Psg*neau001) and the wild soybean ZYD00006 (which showed obvious resistance to *Psg*neau001), through single (one year) self-cross and six instances (six years) of backcross, and the CSSLs consist of 10 generations (6 BC_3_F_5_, 30 BC_3_F_6_, 51 BC_3_F_7_, 2 BC_4_F_4_, 12 BC_4_F_5_, 22 BC_4_F_6_, 13 BC_5_F_4_, 17 BC_5_F_5_, 7 BC_6_F_5_, and 10 BC_6_F_6_) [48]. The final map consisted of 5308 markers associated with 20 linkage groups and was 2655.68 cM long, with an average distance of 0.5 cM between markers [51].

### 4.3. Soybean Planting and Phenotypic Measurement

Three seeds selected from each soybean variety were planted in a greenhouse at 25 °C with a photoperiod of 16 h/8 h (day/night). Plants were inoculated with a *Psg*neau001 suspension at the V1 stage [4]. When the *Psg*neau001 culture at an optical density (600 nm) of 0.8 cultured at 28 °C, it was briefly centrifuged to remove supernatant and was then diluted 1000-fold using 10 mM sterilized MgCl_2_. Silwet-L77 was added to the suspension so that the Silwet-L77concentration was 0.05% [67]. *Psg*neau001 suspension was applied to the back of trifoliate using an atomizer, after which the soybean plants were cultured for 4 days at 100% relative humidity. BSD symptoms were then assessed based on the number of lesions per unit area at 4 days post inoculation according to the following procedure. Three leaf plates were taken from one plant using a hole punch with a diameter of 2 mm and were fully ground in 1.5-mL sterilized tubes containing 400 μL 10 mM MgCl_2_, then added to 600 μL 10 mM MgCl_2_ to 1.0 mL in 1.5 mL tube, diluted 1000 times with 10 mM MgCl_2_. A measure of 100 μL diluted solution was extracted and evenly applied on NYG solid medium with carbenicillin and was then cultured for 2 days. The number of single colonies on the medium was counted and the average was calculated [4]. The phenotypic measurement was performed on three independent samples in each of three independent experiments, and Student’s *t*-test was used for statistical analyses.

### 4.4. QTL Mapping

The cultivated Suinong14 soybean variety and the wild soybean variety ZYD00006 were used to construct chromosome segment substitution lines (CSSLs) using a previously published genetic map [34,48]. BSD-related QTLs were then identified using a WinQTL Cartographer and composite interval mapping techniques were used as follows [47]. The control marker number was 5 and window size was 10 cM. A walk speed of 0.5 cM and the forward regression method were selected. Through a composite interval mapping analysis, the proportion of the variance explained by each particular QTL and the additive effects were analyzed. When LOD score was higher than 3.0, indicating the existence of QTLs for BSD-related genes. The experimental threshold levels for linkage were calculated from 1000 permutations of each genotypic marker against the phenotype in the population. Linkage was reported as significant if the two values for a marker were greater than the critical value at *p* = 0.05.

### 4.5. BSA-seq and Analysis

Through analyses of the BSD symptoms observed in CSSLs, 30 individuals that were extremely resistant to *Psg*neau001 and 30 that were highly susceptible to this pathogen were selected. A Hi-DNAsecure Plant Kit (Tiangen. Co., Beijing, China) was used to isolate DNA from the leaves of these plants, and gDNA concentration and purity was assessed using a Nanodrop 2000C instrument and 1.5% agarose gel electrophoresis. A pooled sequencing library was prepared based on the provided directions, after which 150-bp pair-end sequencing was performed with an Illumina HiSeq 2500 instrument (CA, USA) using standard protocols by Beijing Biomarker Technologies Corporation (http://www.biomarker.com.cn, accessed on 12 December 2021). According to the sequencing results, the association analysis between markers and phenotype was conducted by using the Euclidean distance method. The median + 3SD (median plus three standard deviations) of all locus fitting values was taken as the association threshold for analysis, to determine whether markers and phenotype were closely linked. When the specified threshold was exceeded, a marker was considered to be related to the trait.

### 4.6. ZYD00006 Chromosome Fragment Insertion Analysis

Major QTLs associated with a response to *Psg*neau001 were identified via the analysis of ZYD00006 chromosome insertion in combination with the phenotypic assessment of BSD in the 170 CSSLs.

### 4.7. Major QTL Gene Predictions and Candidate Gene qPCR Analyses

Major BSD-related QTLs were identified via QTL mapping, BSA-seq, and ZYD00006 chromosome fragment insertion analysis. The Williams82 reference genome was used when identifying genes within major QTL regions, with candidate genes being annotated using corresponding annotation information [48].

Patterns of candidate gene expression in Suinong14 and ZYD00006 following *Psg*neau001 inoculation or control treatment were assessed via qPCR. Briefly, leaves harvested at 24 h post inoculation with *Psg*neau001 were snap-frozen with liquid nitrogen and ground to a fine powder in a liquid nitrogen-precooled mortar, after which TRIzol was used to extract total RNA. A PrimeScript™ RT reagent Kit (Takara Biotech Co., Beijing, China) was used to synthesize cDNA, and SuperReal PreMix Color (SYBR Green) (Tiangen Co., Beijing, China) was used for qPCR analyses. Three independent experiments were conducted in soybean plants. *GmUKN1* (*Glyma.12g020500*) served as a normalization control and the 2^−ΔΔCt^ method was used to assess the expression patterns of candidate genes [34].

### 4.8. Haplotype Analyses of Natural Soybean Varieties

Candidate gene haplotype analyses were conducted using 310 natural soybean varieties. Genomic sequences for candidate genes, including both the coding sequence as well as the 3000-bp upstream promoter sequence, were obtained from genomic resequencing for these 310 varieties, with local BLAST analyses then being used to identify any SNPs. Analyses were conducted using Haploview 4.2 (MA, USA) with the Haps Format module, and correlations between *Psg*neau001 resistance and these haplotypes were analyzed using GraphPad Prism 8 [47,51].

## Figures and Tables

**Table 1:** Colony-forming units in CSSL populations inoculated with *Psg*neau001.

Trait	Parents		CSSL Population (*n* = 170)			
	ZYD00006	Suinong14	Mean	SD	Kurtosis	Skewness
Colony-forming units	34 **	680	468.35	173.087	−0.140	−0.360

** indicates *p* ≤ 0.01, and SD indicates standard deviation.

**Table 2:** Main QTLs identified in the CSSL population.

No.	Chr/LG	QTL	Position (Mb)	LOD	R^2^ (%)	ADD	Putative Causal Genes or QTLs Identified in Previous Studies
1	Chr02/D1b	*qbsd-02-1*	1.3	2.92	5	−0.48	
2	Chr08/A2	*qbsd-08-1*	7.8	2.54	2	0.7	
3	Chr08/A2	*qbsd-08-2*	9.3	3.89	3	0.9	
4	Chr08/A2	*qbsd-08-3*	11.1	2.95	1	0.24	
5	Chr09/K	*qbsd-09-1*	16.4	3.42	1	0.27	
6	Chr10/O	*qbsd-10-1*	33.3	3.06	7	1.29	*qXav-10* [4]
7	Chr10/O	*qbsd-10-2*	46.7	4.4	8	0.75	*q10.1* [31]
8	Chr11/B1	*qbsd-11-1*	32.5	2.98	6	0.48	*qSCN3-11* [32]
9	Chr11/B1	*qbsd-11-2*	32.7	3.43	6	0.42	*qSCN3-11* [32]
10	Chr16/J	*qbsd-16-1*	33.2	2.89	2	0.31	*qXav-16* [4]

Note: Chr, chromosome; LG, linkage group all names of the chromosomes’ corresponding linkage groups were derived from the Soybase (https://www.soybase.org/sbt/, accessed on 12 December 2021.); QTL, quantitative trait loci; LOD, log of odds; R^2^, phenotypic variance explained; ADD, the additive effects contributed by the QTL; the LOD score cutoff for major QTLs was determined by permutation tests (1000 times; *p* < 0.05).

**Table 3:** Main QTLs in CSSLs identified via bulked segregant analysis (BSA).

No.	QTL	Chromosome ID	Start Position (bp)	End Position (bp)	Size (Mb)
1	*qbsd-bsa-1*	3	380000	7390000	7.01
2	*qbsd-bsa-2*	10	0	3150000	3.15
3	*qbsd-bsa-3*	10	43910000	48050000	4.14

## Data Availability

Data is contained within the article or supplementary material.

## Supplementary Materials

The following supporting information can be downloaded at: https://www.mdpi.com/article/10.3390/ijms24054618/s1.
