# Supplementary material for: Identifications of QTLs and Candidate Genes Associated with Pseudomonas syringae Responses in Cultivated Soybean (Glycine max) and Wild Soybean (Glycine soja)
Source: Int J Mol Sci. 2023 Feb 27;24(5):4618. doi: 10.3390/ijms24054618 (PMC10003559; doi:10.3390/ijms24054618)
Supplement: Supplementary file 1 [file ijms-24-04618-s001.zip › Supplementary tables.pdf]

Table S1. Colony forming units in 310 soybean natural varieties inoculated with *Psg*neau001

No.	Name	Type	Average of colonies	Grade of disease	Haplotype
1	1020-Ke Jiao 07	Improved cultivar	452.33±41.19	3	Hap1
2	1037-He Nong 75	Improved cultivar	523.33±22.01	3	Hap1
3	75-Ke 732	Improved cultivar	245.33±31.39	2	Hap2
4	Bei 25031	Improved cultivar	854.67±50.65	5	Hap1
5	Bei An 1	Improved cultivar	622.33±31.09	4	Hap1
6	Bei Dou 36	Improved cultivar	323.33±27.74	2	Hap2
7	Bei Dou 40	Improved cultivar	601.67±36.12	4	Hap1
8	Bei Dou 43	Improved cultivar	533.00±38.97	3	Hap1
9	Bei Feng 10	Improved cultivar	356.33±43.02	2	Hap2
10	Bei Feng 11	Improved cultivar	419.00±60.61	3	Hap2
11	Bei Feng 9	Improved cultivar	381.00±52.20	2	Hap2
12	Bei Feng 9-2	Improved cultivar	713.33±88.34	4	Hap1
13	Bei Jiang 1	Improved cultivar	102.33±34.59	1	Hap2
14	Bei Jiang 91	Improved cultivar	658.00±102.43	4	Hap1
15	Bei Jiao 05-8021	Improved cultivar	86.33±27.02	0	Hap2
16	Bei Jiao 9206	Improved cultivar	893.67±113.58	5	Hap1
17	Bei Xing 1	Improved cultivar	475.33±93.68	3	Hap1
18	Bin Dou 10	Improved cultivar	498.67±82.12	3	Hap1
19	BlackJack 21	Improved cultivar	575.67±90.53	3	Hap1
20	Chang Nong 17	Improved cultivar	432.33±68.97	3	Hap2
21	Chang Nong 20	Improved cultivar	476.67±95.38	3	Hap1
22	Chang Nong 29	Improved cultivar	567.67±70.32	3	Hap1
23	Chi Dou 1	Improved cultivar	254.33±52.54	2	Hap2
24	Dong Da 2	Improved cultivar	893.33±82.38	5	Hap1

25	Dong Nong 44	Improved cultivar	869.33±78.29	5	Hap1
26	Dong Nong 50	Improved cultivar	396.33±31.88	2	Hap2
27	Dong Nong 56	Improved cultivar	777.33±61.89	4	Hap1
28	Dong Nong 57	Improved cultivar	353.67±40.65	2	Hap2
29	Dong Nong 76	Improved cultivar	254.67±33.08	2	Hap1
30	Dong Nong 76	Improved cultivar	832.33±91.68	5	Hap2
31	Dong Nong 88	Improved cultivar	365.33±49.52	2	Hap2
32	Dong Nong Dou 253	Improved cultivar	153.67±32.04	1	Hap2
33	Dong Nong L202	Improved cultivar	54.33±10.97	0	Hap2
34	Dong Sheng 1	Improved cultivar	649.33±70.71	4	Hap1
35	Dong Sheng 3	Improved cultivar	321.33±57.71	2	Hap2
36	Dong Sheng 7	Improved cultivar	500.33±68.41	3	Hap1
37	Dong Sheng 92	Improved cultivar	321.33±67.86	2	Hap2
38	Feng Dou 1	Improved cultivar	102.33±24.01	1	Hap2
39	Feng Shou 24	Improved cultivar	406.33±40.72	3	Hap1
40	Feng Shou 25	Improved cultivar	97.67±17.79	0	Hap2
41	Feng Shou 26	Improved cultivar	543.33±76.43	3	Hap1
42	Feng Shou 26	Improved cultivar	752.33±49.86	4	Hap1
43	Feng Shou 5	Improved cultivar	373.33±44.64	2	Hap2
44	Fu Yu Du Lu Dou	Improved cultivar	624.67±96.24	4	Hap1
45	H09-95	Improved cultivar	526.67±70.87	3	Hap1
46	H10-1518	Improved cultivar	616.33±78.21	4	Hap1
47	H10-2430	Improved cultivar	653.67±78.65	4	Hap1
48	H16	Improved cultivar	58.33±13.43	0	Hap2
49	H37	Improved cultivar	933.33±144.24	5	Hap1
50	H38	Improved cultivar	710.33±81.03	4	Hap1

51	H38-2	Improved cultivar	365.33±38.81	2	Hap2
52	H39	Improved cultivar	848.67±109.77	5	Hap1
53	Ha 05-7778	Improved cultivar	320.33±43.13	2	Hap2
54	Ha 06-3869	Improved cultivar	913.67±108.91	5	Hap1
55	Ha 11-3646	Improved cultivar	380.33±39.31	2	Hap2
56	Ha 12-3510	Improved cultivar	555.33±68.09	3	Hap1
57	Ha 12-4547	Improved cultivar	436.67±81.03	3	Hap1
58	Ha 12-4891	Improved cultivar	105.00±27.22	1	Hap2
59	Ha 13-2187	Improved cultivar	308.67±30.24	2	Hap2
60	Ha 13-2413	Improved cultivar	737.33±80.79	4	Hap1
61	Ha 13-2958	Improved cultivar	848.33±105.77	5	Hap1
62	Ha 14-2028	Improved cultivar	343.33±40.07	2	Hap2
63	Ha 14-2146	Improved cultivar	391.33±47.27	2	Hap2
64	Hai 6055	Improved cultivar	745.33±68.65	4	Hap1
65	H-Bei Dou 40	Improved cultivar	604.33±76.03	4	Hap1
66	He 00-23	Improved cultivar	497.33±90.31	3	Hap1
67	He Dou 6	Improved cultivar	197.67±26.27	2	Hap2
68	He Feng 25	Improved cultivar	314.33±59.21	2	Hap2
69	He Feng 29	Improved cultivar	725.33±62.05	4	Hap1
70	He Feng 30	Improved cultivar	395.33±50.29	2	Hap2
71	He Feng 40	Improved cultivar	901.33±88.49	5	Hap1
72	He Feng 47	Improved cultivar	473.33±60.17	3	Hap1
73	He Feng 50	Improved cultivar	895.33±118.03	5	Hap1
74	He Feng 51	Improved cultivar	599.67±51.33	4	Hap1
75	He Feng 55	Improved cultivar	345.33±53.53	2	Hap2
76	He Fu 03-775	Improved cultivar	773.33±93.4	4	Hap1

77	He Fu 05	Improved cultivar	546.67±80.33	3	Hap1
78	He Nan 39	Improved cultivar	419.33±69.24	3	Hap1
79	He Nong 62	Improved cultivar	471.00±69.2	3	Hap1
80	He Nong 73	Improved cultivar	601.33±93.25	4	Hap1
81	He Nong 73-2	Improved cultivar	782.67±72.97	4	Hap1
82	He Nong 75	Improved cultivar	396.33±52.94	2	Hap2
83	He Nong 85	Improved cultivar	714.67±96.00	4	Hap1
84	He Nong 95	Improved cultivar	746.67±88.01	4	Hap1
85	HE14	Improved cultivar	653.33±77.11	4	Hap1
86	Hei He 13	Improved cultivar	159.33±28.29	1	Hap2
87	Hei He 17	Improved cultivar	82.67±19.50	0	Hap2
88	Hei He 18	Improved cultivar	153.33±26.50	1	Hap2
89	Hei He 21	Improved cultivar	492.67±69.62	3	Hap1
90	Hei He 23	Improved cultivar	533.67±60.70	3	Hap1
91	Hei He 33	Improved cultivar	664.33±62.93	4	Hap1
92	Hei HE 38	Improved cultivar	458.33±90.56	3	Hap1
93	Hei He 39	Improved cultivar	660.33±105.98	4	Hap1
94	Hei He 42	Improved cultivar	442.33±54.72	3	Hap1
95	Hei He 45	Improved cultivar	764.67±72.27	4	Hap1
96	Hei He 50	Improved cultivar	584.67±89.29	3	Hap1
97	Hei He 51	Improved cultivar	246.33±38.08	2	Hap2
98	Hei He 52	Improved cultivar	510.67±87.09	3	Hap1
99	Hei He 53	Improved cultivar	502.33±56.89	3	Hap1
100	Hei He 6	Improved cultivar	393.67±58.11	2	Hap2
101	Hei Jiao 1-1161	Improved cultivar	256.33±52.92	2	Hap2
102	Hei Kang 06-7	Improved cultivar	774.33±66.64	4	Hap1

103	Hei Nong 35	Improved cultivar	877.33±82.92	5	Hap1
104	Hei Nong 50	Improved cultivar	895.33±118.45	5	Hap1
105	Hei Nong 56	Improved cultivar	584.33±121.08	3	Hap1
106	Hei Nong 75	Improved cultivar	487.33±69.76	3	Hap1
107	Hei Sha 45	Improved cultivar	813.33±77.03	5	Hap1
108	Heng Dou 15	Improved cultivar	702.33±77.78	4	Hap1
109	Holt	Improved cultivar	566.33±121.06	3	Hap1
110	Hu Lin 1	Improved cultivar	147.33±19.86	1	Hap2
111	Hua Jiang 12	Improved cultivar	623.33±93.87	4	Hap1
112	Hua Jiang 2	Improved cultivar	394.67±47.25	2	Hap2
113	Hua Jiang 4	Improved cultivar	353.67±44.23	2	Hap2
114	Japan 1	Improved cultivar	421.67±86.56	3	Hap1
115	Ji 101	Improved cultivar	527.67±102.08	3	Hap1
116	Ji 94	Improved cultivar	406.67±126.14	3	Hap1
117	Ji Hei 6	Improved cultivar	967.33±117.41	5	Hap1
118	Ji Ke 1	Improved cultivar	366±41.51	2	Hap2
119	Ji Lin Xiao Li Dou 7	Improved cultivar	734.33±90.18	4	Hap1
120	Ji Nong 18	Improved cultivar	731.33±89.05	4	Hap1
121	Ji Yu 105	Improved cultivar	412.00±64.82	3	Hap1
122	Ji Yu 108	Improved cultivar	702.33±99.67	4	Hap1
123	Ji Yu 109	Improved cultivar	595.33±66.71	3	Hap1
124	Ji Yu 67	Improved cultivar	659.00±121.01	4	Hap1
125	Ji Yu 72	Improved cultivar	487.33±65.29	3	Hap1
126	Jia Dou 36	Improved cultivar	638.00±83.86	4	Hap1
127	Jia Nong 1	Improved cultivar	729.33±79.98	4	Hap1
128	Jia Nong 2	Improved cultivar	708.00±104.48	4	Hap1

129	Jiang Mo Dou 1	Improved cultivar	320.00±48.82	2	Hap2
130	Jiang Nong 416	Improved cultivar	745.00±87.57	4	Hap1
131	Jiang Nong 417	Improved cultivar	497.33±61.70	3	Hap1
132	Jim	Improved cultivar	515.00±92.07	3	Hap1
133	Jin Feng 55-2	Improved cultivar	520.33±96.86	3	Hap1
134	Jin Lin 30	Improved cultivar	495.33±97.45	3	Hap1
135	Jin Pin 42	Improved cultivar	369.00±39.89	2	Hap2
136	Jin San 14-70	Improved cultivar	871.00±125.37	5	Hap1
137	Jin Yuan 55	Improved cultivar	568.33±81.00	3	Hap1
138	Jin Yuan 73	Improved cultivar	673.00±111.72	4	Hap1
139	Jin Yuan 95	Improved cultivar	376.00±58.81	2	Hap2
140	Jiu Feng 10	Improved cultivar	326.67±44.00	2	Hap2
141	Jiu San 14-70	Improved cultivar	642.67±103.73	4	Hap1
142	Jiu San Hei 05-59	Improved cultivar	369.33±49.65	2	Hap2
143	Kariyutaka	Improved cultivar	702.67±83.93	4	Hap1
144	Ke 11-1669	Improved cultivar	456.00±99.96	3	Hap1
145	Ke 732	Improved cultivar	353.33±60.93	2	Hap2
146	Ke C14-732	Improved cultivar	353.67±43.84	2	Hap2
147	Ke Jiao 07	Improved cultivar	60.33±23.46	0	Hap2
148	Ke Jiao 09	Improved cultivar	795.67±85.93	4	Hap1
149	Ke Shan 1	Improved cultivar	97.67±17.01	0	Hap2
150	Ken 05-3762	Improved cultivar	482.33±88.08	3	Hap1
151	Ken 09-1723	Improved cultivar	439.00±65.78	3	Hap1
152	Ken Feng 16	Improved cultivar	481.00±81.73	3	Hap1
153	Ken Feng 22	Improved cultivar	565.67±61.40	3	Hap1
154	Ken Feng 28	Improved cultivar	792.00±51.08	4	Hap1

155	Ken Feng 7	Improved cultivar	582.33±41.67	3	Hap1
156	Ken Jian 28	Improved cultivar	642.00±31.97	4	Hap1
157	Ken K11-7456	Improved cultivar	691.00±47.53	4	Hap1
158	Ken Nong18	Improved cultivar	382.67±21.82	2	Hap2
159	Kovean	Improved cultivar	641.33±71.13	4	Hap1
160	L05-144	Improved cultivar	589.33±60.01	3	Hap1
161	L65-1274	Improved cultivar	456.00±36.89	3	Hap1
162	L65-34	Improved cultivar	565.33±62.53	3	Hap1
163	L65-540	Improved cultivar	576.00±23.55	3	Hap1
164	L67-166	Improved cultivar	258.33±20.50	2	Hap2
165	L67-971	Improved cultivar	635.67±82.87	4	Hap1
166	L72-1241	Improved cultivar	533.00±33.26	3	Hap1
167	L72D-4045	Improved cultivar	514.67±24.36	3	Hap1
168	L73-79	Improved cultivar	555.33±19.29	3	Hap1
169	L83-4387	Improved cultivar	181.33±11.90	1	Hap2
170	L85-144	Improved cultivar	684.00±38.18	4	Hap1
171	LG2016	Improved cultivar	280.67±15.63	2	Hap2
172	LG296-7	Improved cultivar	347.00±16.39	2	Hap2
173	Liao 08024	Improved cultivar	675.00±44.21	4	Hap1
174	Liao 08Q104	Improved cultivar	440.00±39.61	3	Hap1
175	Liao Dou 37	Improved cultivar	640.00±19.25	4	Hap1
176	Liao Xiao Li Dou 2	Improved cultivar	330.00±19.51	2	Hap2
177	Long Ken 332	Improved cultivar	505.00±39.90	3	Hap1
178	Long Ken 381	Improved cultivar	418.67±20.73	3	Hap1
179	Long Pin 12-328	Improved cultivar	459.33±23.30	3	Hap1
180	Long Pin 13-369	Improved cultivar	331.00±27.58	2	Hap2

181	Long Qing Dou 2	Improved cultivar	464.00±16.57	3	Hap1
182	M39	Improved cultivar	460.33±26.84	3	Hap1
183	Magnolid	Improved cultivar	341.33±18.80	2	Hap2
184	Meng Dou 9	Improved cultivar	400.67±34.84	3	Hap1
185	Mu 05-026	Improved cultivar	342.67±47.99	2	Hap2
186	Nan Nong 415	Improved cultivar	628.33±31.84	4	Hap1
187	Nen Feng 20	Improved cultivar	478.00±20.61	3	Hap1
188	NO.10	Improved cultivar	477.00±49.50	3	Hap1
189	No.16	Improved cultivar	683.33±35.26	4	Hap1
190	Nong 75	Improved cultivar	222.00±25.47	2	Hap2
191	NT 04	Improved cultivar	397.33±15.28	2	Hap2
192	NT 12	Improved cultivar	557.67±39.84	3	Hap1
193	PI317334B	Improved cultivar	464.33±26.95	3	Hap1
194	POLUKULTURNAYA	Improved cultivar	411.33±18.52	3	Hap1
195	Sheng dou 42	Improved cultivar	832.00±63.60	5	Hap1
196	Sheng Dou 43	Improved cultivar	753.33±42.52	4	Hap1
197	Sheng Dou 48	Improved cultivar	808.33±59.89	5	Hap1
198	Shu Jin 05-9238	Improved cultivar	498.33±51.91	3	Hap1
199	Shuang 302	Improved cultivar	598.33±22.84	3	Hap1
200	Sui 07-1077	Improved cultivar	245.00±16.06	2	Hap2
201	Sui Nong 10	Improved cultivar	428.67±21.08	3	Hap1
202	Sui Nong 13	Improved cultivar	603.67±16.78	4	Hap1
203	Sui Nong 14	Improved cultivar	680.00±42.93	4	Hap1
204	Sui Nong 17	Improved cultivar	327.00±24.59	2	Hap2
205	Sui Nong 18	Improved cultivar	702.00±46.18	4	Hap1
206	Sui Nong 19	Improved cultivar	482.67±24.85	3	Hap1

207	Sui Nong 22	Improved cultivar	328.00±11.78	2	Hap2
208	Sui Nong 26	Improved cultivar	420.67±17.44	3	Hap1
209	Sui Nong 27	Improved cultivar	578.00±22.63	3	Hap1
210	Sui Nong 4	Improved cultivar	488.00±43.73	3	Hap1
211	Sui Nong 40	Improved cultivar	771.67±51.41	4	Hap1
212	Sui Nong 43	Improved cultivar	724.00±49.24	4	Hap1
213	Sui Nong 49	Improved cultivar	129.33±12.28	1	Hap2
214	Sui Nong 50	Improved cultivar	580.67±40.74	3	Hap1
215	Sui Nong 52	Improved cultivar	774.00±35.69	4	Hap1
216	Sui Nong 67	Improved cultivar	777.00±50.62	4	Hap1
217	Sui Nong 68	Improved cultivar	449.00±39.56	3	Hap1
218	Sui Nong 69	Improved cultivar	309.00±27.90	2	Hap2
219	Sui Nong 75	Improved cultivar	636.00±41.77	4	Hap1
220	Sui Nong 76	Improved cultivar	879.33±69.43	5	Hap1
221	Sui Nong 9	Improved cultivar	732.67±35.49	4	Hap1
222	Vinton 81	Improved cultivar	615.00±30.74	4	Hap1
223	Wu Xing Dou 2	Improved cultivar	612.00±56.50	4	Hap1
224	Wu Xing Dou 3	Improved cultivar	467.00±30.82	3	Hap4
225	Xing Nong 12	Improved cultivar	461.33±43.93	3	Hap4
226	Zhong Huang 10	Improved cultivar	609.00±28.25	4	Hap1
227	Zhong Huang 35	Improved cultivar	770.33±77.59	4	Hap1
228	Zhong Huang 688	Improved cultivar	470.00±12.08	3	Hap4
229	Zi Hua 2	Improved cultivar	370.00±23.79	2	Hap4
230	777	Landrace	154.33±10.21	1	Hap1
231	1303	Landrace	455.67±17.25	3	Hap1
232	1367	Landrace	309.67±33.49	2	Hap1

233	H914 WJH	Landrace	224.33±23.61	2	Hap1
234	Tie Jia Zi (I)	Landrace	698.00±45.31	4	Hap1
235	Da Li Huang	Landrace	392.33±29.80	2	Hap1
236	Gong Ye 03-557	Landrace	498.33±20.53	3	Hap3
237	Gong Ye 03-5570	Landrace	248.33±30.66	2	Hap1
238	Gong Ye 04L-141	Landrace	291.33±15.92	2	Hap1
239	Hei Qi	Landrace	723.33±18.62	4	Hap3
240	Hei Tie Jia	Landrace	370.33±20.98	2	Hap1
241	Hu Lin Lao Tai Tai Tiao	Landrace	394.67±25.00	2	Hap2
242	Hua Dian Zi Hua Shao Tiao Dou	Landrace	464.67±18.12	3	Hap3
243	Hua Nan Xiao Huang Jin	Landrace	806.00±81.58	5	Hap3
244	Hua Nan Yi Wo Feng	Landrace	304.67±42.87	2	Hap1
245	Huai De Bai Hua Da Li	Landrace	247.33±13.27	2	Hap2
246	Huan Hua Si Li Huang	Landrace	925.00±65.43	5	Hap3
247	J-LG2016-1	Landrace	256.67±20.50	2	Hap2
248	Man Cang Jin	Landrace	953.00±72.97	5	Hap3
249	Man Jin Cang	Landrace	343.00±24.54	2	Hap1
250	Mao Yan Dou	Landrace	281.33±21.30	2	Hap2
251	Mo Shi 1	Landrace	153.67±17.15	1	Hap2
252	Mo Shi 9	Landrace	256.67±12.76	2	Hap2
253	N010	Landrace	141.67±17.33	1	Hap2
254	Nattosan	Landrace	334.67±26.54	2	Hap2
255	Nong An Jiang Se Dou	Landrace	339.67±17.99	2	Hap1
256	Pan Shi Dou	Landrace	214.67±18.62	2	Hap2
257	PSB576	Landrace	270.67±22.45	2	Hap2
258	q15	Landrace	656.67±48.61	4	Hap3

259	Qian Guo Zi Hua Lan Qi	Landrace	227.33±29.04	2	Hap1
260	Qing An Hei Dou	Landrace	315.67±38.47	2	Hap2
261	Qing An Xiao Huang Jin	Landrace	295.00±10.61	2	Hap1
262	Qing Da Dou	Landrace	93.67±9.29	0	Hap2
263	Qing Gang Si Li Ding	Landrace	314.00±23.51	2	Hap2
264	Qing Pi	Landrace	498.00±20.05	3	Hap3
265	Shen Nen	Landrace	57.00±6.98	0	Hap2
266	Shu Lan Man Cang He	Landrace	247.67±19.70	2	Hap2
267	Shuang Liao Cha Dou	Landrace	304.33±12.71	2	Hap2
268	Shuang Se Dou	Landrace	321.67±20.07	2	Hap1
269	Shuang Yang Zao Huang Dou	Landrace	58.67±2.36	0	Hap2
270	Si Li Huang	Landrace	238.33±17.00	2	Hap2
271	SS202	Landrace	328.67±20.95	2	Hap1
272	Sun Hao Da Bai Shou Mei	Landrace	278.00±15.90	2	Hap2
273	Suo Yi Ling	Landrace	689.00±47.38	4	Hap3
274	T219H	Landrace	396.67±39.60	2	Hap1
275	Tai Lai Si Li Huang	Landrace	455.67±18.80	3	Hap3
276	Tian E Dan	Landrace	304.00±22.76	2	Hap1
277	Tie Jia Qing	Landrace	229.33±6.85	2	Hap1
278	Tie Jia Si Li Huang	Landrace	486.00±28.61	3	Hap3
279	Tie Jia Zi (II)	Landrace	302.00±19.60	2	Hap1
280	Tong Hua Ping Di Huang	Landrace	238.00±11.43	2	Hap1
281	Wang Qing Ba RenGou	Landrace	194.67±14.82	1	Hap1
282	Wang Qing Ji Guan	Landrace	310.67±18.15	2	Hap1
283	Wo Dou	Landrace	316.33±10.87	2	Hap1
284	Wu Chang Dou	Landrace	456.33±22.84	3	Hap3

285	Xiao Bai Dou	Landrace	250.67±27.01	2	Hap3
286	Xiao Bai Qi	Landrace	367.33±30.65	2	Hap1
287	Xiao Huang Dou	Landrace	457.67±32.40	3	Hap4
288	Ya Po Che	Landrace	219.67±7.72	2	Hap3
289	Yang Huang Du	Landrace	214.00±13.14	2	Hap3
290	Yong Feng Dou	Landrace	298.33±20.42	2	Hap3
291	Yong Ji Zao Dou	Landrace	252.67±9.81	2	Hap3
292	You Hu Dou	Landrace	409.33±25.49	3	Hap4
293	Yu Shu Chang Cha Dou	Landrace	339.00±16.57	2	Hap3
294	Yuan Bao Jin	Landrace	354.67±11.09	2	Hap3
295	Zao Dou	Landrace	469.33±38.85	3	Hap4
296	Zhang Bao Da Dou	Landrace	359.67±36.01	2	Hap4
297	Zhang De Heng Da Dou	Landrace	174.00±8.60	1	Hap4
298	Zhao Yuan Zi Hua Cuo Zi	Landrace	222.67±7.76	2	Hap4
299	Zhao Zhou Xiao Li Huang	Landrace	252.67±8.65	2	Hap4
300	Zi Hua Cuo Zi	Landrace	29.00±2.16	0	Hap4
301	ZYD00006	wild (*G. soja*)	33.67±3.40	0	Hap2
302	ZYD0088	wild (*G. soja*)	178.33±10.14	1	Hap1
303	ZYD0097	wild (*G. soja*)	28.33±2.05	0	Hap2
304	ZYD0118	wild (*G. soja*)	261.67±21.23	2	Hap3
305	ZYD0170	wild (*G. soja*)	166.67±25.38	1	Hap2
306	ZYD0371	wild (*G. soja*)	112.33±7.13	1	Hap2
307	ZYD0386	wild (*G. soja*)	284.00±30.59	2	Hap3
308	ZYD0694	wild (*G. soja*)	223.00±20.85	2	Hap4
309	ZYD6798	wild (*G. soja*)	294.33±19.43	2	Hap4
310	ZYD6832	wild (*G. soja*)	424.67±28.00	3	Hap4

Table S2. Gene models in the QTL for BSD on chromosome 10

No.	Gene ID	Extron	Intron	Promoter region	Annotation
1	*Glyma.10G228300*	0	0	0	Protein of unknown function
2	*Glyma.10G228400*	0	4	0	Protein of unknown function
3	*Glyma.10G228500*	1	1	5	Protein of unknown function
4	*Glyma.10G228600*	11	3	3	Leucine Rich Repeat (LRR_1)
5	*Glyma.10G228700*	0	7	0	Uncharacterized conserved protein
6	*Glyma.10G228800*	19	6	0	Leucine Rich Repeat (LRR_1)
7	*Glyma.10G228900*	1	3	2	Protein kinase domain (Pkinase)
8	*Glyma.10G229000*	3	7	6	CAAX AMINO TERMINAL PROTEASE FAMILY PROTEIN
9	*Glyma.10G229100*	0	0	0	Protein of unknown function
10	*Glyma.10G229200*	1	0	12	MEMBRANE-ASSOCIATED KINASE REGULATOR 6-RELATED
11	*Glyma.10G229300*	7	11	5	S-TYPE ANION CHANNEL SLAH2-RELATED
12	*Glyma.10G229400*	3	11	5	AAA ATPASE
13	*Glyma.10G229500*	1	12	3	F-BOX FAMILY PROTEIN-RELATED
14	*Glyma.10G229600*	10	27	1	RETICULON-LIKE PROTEIN B4
15	*Glyma.10G229700*	0	1	0	Protein of unknown function
16	*Glyma.10G229800*	7	1	4	MEMBRANE-ASSOCIATED KINASE REGULATOR 5-RELATED
17	*Glyma.10G229900*	0	14	0	Predicted transporter/transmembrane protein
18	*Glyma.10G230000*	2	7	3	PROTEIN SGT1 HOMOLOG A-RELATED
19	*Glyma.10G230100*	8	2	1	PPR repeat (PPR)
20	*Glyma.10G230200*	2	1	15	WRKY TRANSCRIPTION FACTOR 27-RELATED
21	*Glyma.10G230300*	6	2	4	Thiomorpholine-carboxylate dehydrogenase
22	*Glyma.10G230400*	1	1	0	Domain of unknown function (DUF4228) (DUF4228)
23	*Glyma.10G230500*	0	0	0	COPPER TRANSPORT FAMILY PROTEIN-RELATED

24	*Glyma.10G230600*	1	3	1	COPPER TRANSPORT FAMILY PROTEIN-RELATED
25	*Glyma.10G230700*	2	7	0	PROTEIN REVEILLE 3-RELATED
26	*Glyma.10G230800*	3	2	2	Protein of unknown function
27	*Glyma.10G230900*	1	0	1	ENDO-POLYGALACTURONASE-LIKE PROTEIN-RELATED
28	*Glyma.10G231000*	2	0	7	ENDO-POLYGALACTURONASE-LIKE PROTEIN-RELATED
29	*Glyma.10G231100*	8	9	0	ENDO-POLYGALACTURONASE-LIKE PROTEIN-RELATED
30	*Glyma.10G231200*	2	5	4	BETA-CAROTENE 3-HYDROXYLASE 1
31	*Glyma.10G231300*	1	0	4	Protein of unknown function
32	*Glyma.10G231400*	1	6	0	Uncharacterized conserved protein
33	*Glyma.10G231500*	2	0	0	Protein tyrosine kinase (Pkinase_Tyr)
34	*Glyma.10G231600*	14	15	0	FERRIC REDUCTION OXIDASE 4-RELATED
35	*Glyma.10G231700*	0	2	0	NADPH OXIDASE
36	*Glyma.10G231800*	0	0	0	MATE EFFLUX FAMILY PROTEIN
37	*Glyma.10G231900*	0	0	0	Protein of unknown function
38	*Glyma.10G232000*	3	1	0	SCARECROW-LIKE PROTEIN 8
39	*Glyma.10G232100*	3	0	0	Protein of unknown function
40	*Glyma.10G232200*	1	3	0	MATE EFFLUX FAMILY PROTEIN

Table S3. Primers used in this study

No.	Name of primer	Sequence 5’-3’	Annotation and Functions
1	*16S rDNA*	Fwd-AGAGTTTGATCCTGGCTCAG<br>Rev-TACGGCTACCTTGTTACGACTT	Clone of *16S rDNA*
2	q*Glyma.10G228500*	Fwd-TTAACCGTAGTGTAAGTCTTGCT<br>Rev-CCCCTCCAAACACTTAATCACC	qRT-PCR
3	q*Glyma.10G228600*	Fwd-GCGACTTTGTGATTCGAAAGAT<br>Rev-TCTGTGTGAAGTAACTTGTCGA	qRT-PCR
4	q*Glyma.10G228800*	Fwd-TCCAAAATTACAGCGCTTGTAC<br>Rev-CAATGCGTAGAAGTTGAAACGA	qRT-PCR
5	q*Glyma.10G228900*	Fwd-CATTAATATGCCACCAATGCCA<br>Rev-GTCAACTGTTACATGTTCGTCC	qRT-PCR
6	q*Glyma.10G229000*	Fwd-GCACACTGAAACAATTAACCCT<br>Rev-TTTTTAGGAAACGTGACTGCTG	qRT-PCR
7	q*Glyma.10G229200*	Fwd-AGAACATTGTCAAGGAGAGTGT<br>Rev-TCAGCAGAAAAGGCTACACTAA	qRT-PCR
8	q*Glyma.10G229300*	Fwd-GAAACTTCTGCTTCCTGAGTTG<br>Rev-ACGGTTAAGGTTGTTTTCCATG	qRT-PCR
9	q*Glyma.10G229400*	Fwd-CTATTTTTGGTTTCCCGCATGA<br>Rev-TGTTCTTTGGGTTGATCTGTTG	qRT-PCR
10	q*Glyma.10G229500*	Fwd-ATCGAGAAGCAAGTTTTATGGC<br>Rev-CCAACAACAACCACATCATCAT	qRT-PCR
11	q*Glyma.10G229600*	Fwd-ATAAAAGTCCACCTAGTCCTGC<br>Rev-GTACACGTTCATCGAGTCAATG	qRT-PCR
12	q*Glyma.10G229800*	Fwd-GAACTACATTGCTGGAACTTCG	qRT-PCR

		Rev-CGAGTACTCGTTGATGAGAAGA	
13	q*Glyma.10G230000*	Fwd-TGCACAGAGGTTTAGTGTTTTG	qRT-PCR
		Rev-GCTTCAAGCTTATCCCAATCTG	
14	q*Glyma.10G230100*	Fwd-CAGTGTTCATGTAGAGGTCACT	qRT-PCR
		Rev-ACATTGCTAGAACCACGAGTAA	
15	q*Glyma.10G230200*	Fwd-GGTGACCGTTGGATTATGATTG	qRT-PCR
		Rev-GAAGATTCAAGTGTACGGCTTC	
16	q*Glyma.10G230300*	Fwd-CTCACCCTTTACAGAACCTCAT	qRT-PCR
		Rev-TTCCCCACTTTGACCAAATTTG	
17	q*Glyma.10G230400*	Fwd-TTTCCCGCTCTATAATTCCCTC	qRT-PCR
		Rev-GCACTGAATTCCATGATACCAC	
18	q*Glyma.10G230600*	Fwd-GTTGTGTTGAAGGTGGAAGTAC	qRT-PCR
		Rev-TCTTGTCTTTCATGTCCACAGA	
19	q*Glyma.10G230700*	Fwd-CTATAGATGTTGAGACGGTGCT	qRT-PCR
		Rev-TAAGTAGCACTCTTTAACCGCA	
20	q*Glyma.10G230800*	Fwd-ACCTCTCCTAGTTACAGTCAGA	qRT-PCR
		Rev-TGTCAGTTACTCTTACAAGCGT	
21	q*Glyma.10G230900*	Fwd-TGGCATAACATCCAACACAAAG	qRT-PCR
		Rev-CCTGAGATGCAAGAATAACAGC	
22	q*Glyma.10G231000*	Fwd-AATAAAAGGGCTTACCATTCTCG	qRT-PCR
		Rev-TACTGCCACACAATCATCACC	
23	q*Glyma.10G231100*	Fwd-AATTGAATCTCACCAGGCCAT	qRT-PCR
		Rev-GCCCTTTTATTGTTATGTTACTGCT	
24	q*Glyma.10G231200*	Fwd-GATCAAAACGGTCGAACAAGAA	qRT-PCR
		Rev-AACGAGATAAGTGAACCTCTGG	

25	q*Glyma.10G231300*	Fwd-CTACTTCTGCTGCCATCATTTC<br>Rev-CATGAAGAGGCCTAGCTTATGA	qRT-PCR
26	q*Glyma.10G231400*	Fwd-GCAACCACTTCTGTGATGAATT<br>Rev-CATTCTCAACCCTTTCCCTAGT	qRT-PCR
27	q*Glyma.10G231500*	Fwd-GATTTGTCAGCCCTTACAATCC<br>Rev-GCACAACATTAGTACCGACATC	qRT-PCR
28	q*Glyma.10G231600*	Fwd-AATTGCATTAGTGATGTGGGTG<br>Rev-AGAGAGTGTAGAGATGGTGAGT	qRT-PCR
29	q*Glyma.10G232000*	Fwd-GAACTTGAAGCGAGTCAGGATC<br>Rev-CTTTGATCGAATCAGCGACTC	qRT-PCR
30	q*Glyma.10G232100*	Fwd-GTCCGAAAGAGTAGCAGAATGA<br>Rev-GGGAGAAGTACAATCTAAGGGG	qRT-PCR
31	q*Glyma.10G232200*	Fwd-TACGTGTACATTCTACGTTCGT<br>Rev-GATTCGACGTTAAACTCTCACG	qRT-PCR
32	q*GmUKN1*	Fwd-TGGTGCTGCCGCTATTTACTG<br>Rev-GGTGGAAGGAACTGCTAACAATC	Reference gene for qRT-PCR

Table S4. Promoter sequence of different haplotypes

Type	Sequence
Hap1	ATGCATACTAGAACTAAACTTTCTAAAACTCCCAAATTCATATAACTAAGGGTGATCAAATTGGGTCGTTGTTTATCACG<br>CATTCACAGAAAGTATGATTTTTTTATTTAAATGTTTGCTATAAGAAAATATATCGTTTGCATATAATTTAGACTGCTTC<br>CTATTTTGAAAGTGTAAAGTCGATAAACGTTTTATAAGTGTTTTATATGTAGGAAGAGTGCAAAGTTTGAGCATGTGAAA<br>TGCTACAAATTGTGCAAAATTGGCAATGGTGATCATATTTTATTTACACAACATTTTTCAATCTCAACTACTATTGTTCT<br>TCCATTAGTTTCACTTCCATGCTATTTCTTAATGTCAATCTCAAATTAATTTTTTTTGTCACATATACAAGTATCAAATT<br>AAGTATAACAACATATAAATAAAAAAGATTCACAAACCTAATGTCTTGAGATTTTTTATTAAAGCATGATGTCAAATTTA<br>GTTATATAATATTTTTGTGTCCTATTATTAATGAAATCTCCTAGAATCTTATTCAAGTATTTGATGAGGAGTTCTAGAAG<br>ACAAACTCATTGACAACTTCAACTAGATTTATTATTAAAATATATTTGTATATAAAAATAAAAAAAATTCCCTATTTATA<br>TAATGCTTTTGTATTTCTCATGAATTTAACTTTTAGAGATAAATAAACCAAATTTGCAATATTTTTAATATTTCTATTGT<br>TTCAAAATTAAAACATGTATGAATTGCAATTTCAATTTAAAATAAAATAAAAATATAAAAAAAGTTGGATTCACTCTTAG<br>ATCCACTAAAAAAAGTTACTAATCCAAGTTAATTTCTACCATTAAAGTAAAAATTACAGGTGTTCTTAAATATTACAATA<br>TATGATAACAAAAGACCCACTAAAAAAAATTAAGAGCTTAAATTAAGTTCTTGCTTTCGAGATAATATGTGTTTGTATTT<br>ATGGTAGAACTACCATAGCATGAAATCCCATATTATTTCACCACATAAGTATCAAAGTTGATACTTTTAAAAACGTAAAT<br>TGGATTGTTGTTATTGGCAATCTAAACATACACATACTCAATTTTGAAATGTTATTTTTGTTGTAATTACTGTTATTAGT<br>TTTTAGTTAGGTTTCGAATAGAACCTTAGAATATCTTCACAAACTTTTTATCATTTGCTTTGGGCTAGTTATAATAATCA<br>TACTTAAAATTTTTTCGTTGCCTTCTCTCTCAAAATGTAATCCACATAAATTGAATATAGATTTTTTTTCCCCCGCAAAA<br>TACCCACCCATATCCTTGAAATATTATTTGAGTGCGACTTCGACCATCTTAAAATATTTCAATTTGTTGTTAAACTTCGC<br>ATCTATGCATTATTGTTTAATTGTGATTTTCTTTTGTCACATTCCACATATTAGAATTCAAATTTAAATCCACCTCTGCA<br>GTTCTACTTCTTGCTTTGTTCAACTCCAACGGTCCTACCACATACCATTATTATTAGTAGTAGTAATAATAGTTTAACTT<br>ACATACATTATTAATGTAAAACATATTTATACATACACTCGTTATTAATTAATTTCCACCTATGCATTGATATAAAGTGA

Hap2	GGATAAAGTAAATTTGTATTACATATCCATGTAAGTTTAATTTATAATAACTATATAATTATATATAAATTAAATTCTAT<br>TATATTGATTACATCTTGTACATGTTTGTTTTATTTTCAGAATCCATTCCTTCCACCATGCATGTTTCAACGTAAGATTT<br>GTTATTGATAGTATTAATTTTTTTTTTTTGTTGACGGGATAGTATTGATTTTTAATTACGTTTATTTTGTATTAACTTTA<br>AAATAAAAGAACAAAGCCAGCTTAATTTTTAACACTTTCGTATTTGGATAAAAACACAAAACAAAACATTTCGGGTCCTT<br>TACTTCTGTTTGAGATCAACGTAATGCAACATTTAAAATTTGTAATTGGGAATTCAAACCTAGTCTAAGTTTAAAATTAA<br>TTTTGAATGACTTAACTGTACAATTCAATACAATACAATGCGCGACAAAATGGAAGACACGTATCAAAGAAATTCCACAC<br>CCGTTATTGCAGACATTTTTGTTATAATAATACAGGTTTGCTATCTACACATTAATGACGCACAACTATTTAGTTTCTTA<br>AAAAGACCAAGTGGCAATGTCTATCTACAAAAATTGTTTATTCAAAAAATCAATCGCCGAAAATAATAATCTTTACAAAT<br>TTGCATTTGCCAAAACCTAAGTACTAATTCCACGTTGAATGTCATGCAGAACCGAAGAACATGGCGGGGTGAGTGTATCT<br>TTAGAGAAATCTGATATAAAATTACGTTTAAAACCAAAATAATTTTATCAATCAGGATTTTTAATTTTAAGTTCATTTTA<br>TTTTTATTCCATAGATTTATAATTTTCAATCGTAAAAAATAAGTTGAATGCTTAATTAACAACACTGGACACAAGGGCGC<br>GTCAGCACCTTCAGTTGATGCACTACCCAATCAGATCCCAATGTTGTTTGTTAACAACTGTTAGAGTTGGAACTTTCCCA<br>CTCAGGGCATCAGAATCATCATCACCCTTAACTTAGGGCCCCCCTTTGACATTTTCACAATGTCAATGAAAAAGCCAAGA<br>GAGAAAGAGAGGGAGAGCATATCTTTCTAACTCCTCCGACCCCTACAAAATTCCACTTTCACATATCTCTTTCTTTTTTT<br>CTCTTTTCAATTTTTCTAGTATAAAATAATTAATTGCCCAACTCCCTCAGTGCTCCACCAACCCCTCTTGCTTCTTCCTC<br>TTATCTTTCTCTCTAGATTTTGCCATTCACCCTAAAAGTCAGCGCCATCTTTTGTGCCTTATTTCCTCCCTCAGTTCCGA<br>AACTTTGTTGCTTTAATTGGGAAAAGAAAAGAAAAACCACACTGATTTTTATTTTTTTATCTTCCTTAATTCATAGCCTT<br>ACCCAGAAGTCTCTTTCTGCTTTTCTCTCCCTCTGCATA<br>ATGCATACTAGAACTAAACTTTCTAAAACTCCCAAATTCATATAACTAAGGGTGATCAAATTGGGTCGTTGTTTATCACG<br>CATTCACAGAAAGTATGATTTTTTTATTTAAATGTTTGCTATAAGAAAATATATCGTTTGCATATAATTTAGACTGCTTC<br>CTATTTTGAAAGTGTAAAGTCGATAAACGTTTTATAAGTGTTTTATATGTAGGAAGAGTGCAAAGTTTGAGCATGTGAAA<br>TGCTACAAATTGTGCAAAATTGGCAATGGTGATCATATTTTATTTACACAACATTTTTCAATCTCAACTACTATTGTTCT<br>TCCATTAGTTTCACTTCCATGCTATTTCTTAATGTCAATCTCAAATTAATTTTTTTTGTCACATATACAAGTATCAAATT<br>AAGTAGAACAACATATAAATAAAAAAGATTCACAAACCTAATGTCTTGAGATTTTTTATTAAAGCATGATGTCAAATTTA<br>GTTATATAATATTTTTGTGTCCTATTATTAATGAAATCTCCTAGAATCTTATTCAAGTATTTGATGAGGAGTTATAGAAG<br>ACAAACTCATTGACAACTTCAACTAGATTTATTATTAAAATATATTTGTATATAAAAATAAAAATAAAAATAATTTCCCT

ATTTATATAATGCTTTTGTATTTCTCATGAATTTATCTTTTAGAGATAAATAAACCAAATTTGCAATATTTTTAATATTT
CTATTGTTTCAAAATTAAAACATGTATGAATTACAATTTCAATTTAAAATAAAATAAAAATATAAAAAAAGTTGGATTCA
CTCTTAGATCCACTAAAAAAAGTTACTAATCCAAGTTAATTTCTACCATTAAAGTAAAAATTACAGGTGTTCTTAAATAT
TACAATATATGATAACAAAAGACCCACTAAAAAAAATTAAGAGCTTAAATTAAGTTCTTGCTTTCGAGATAATATGTGTT
TGTATTTATGGTAGAACTACCATAGCATGAAATCCCATATTATTTCACCACATAAGTATTAAAGTTGATACTTTTAAAAA
CGTAAATTGGATTGTTGTTATTGGCAATCTAAACATACACATACTCAATTTTGAAATGTTATTTTTGTTGTAATTACTGT
TATTAGTTTTTAGTTAGGTTTCGAATAGAACCTTAGAATATCTTCACAAACTTTTTATCATTTGCTTTGGGCTAGTTATA
ATAATCATACTTAAAAGTTTTTCGTTGCCTTCTCTCTCAAAATGTAATCCACATAAATTGAATATAGATTTTTTTCCCCG
CAAAATACCCACCCATATCCTTGAAATATTATTTGAGTGCGACTTCGACCATCTTAAAATATTTCAATTTGTTGTTAAAC
TTCGCATCTATGCATTATTGTTTAATTGTGATTTTCTTTTGTCACATTCCACATATTAGAATTCAAATTTAAATCCACCT
CTGCAGTTCTACTTCTTGCTTTGTTCAACTCCAACGGTCCTACCACATACCATTATTATTAGTAGTAGTAATAATAGTTT
AACTTACATACATTATTAATGTAAAACATATTTATACATACACTCGTTATTAATTAATTTCCACCTATGCATTGATATAA
AGTGAGGATAAAGTAAATTTGTATTACATATCCATGTAAGTTTAATTTATAATAACTATATAATTATATATAAATTAAAT
TCTATTATATTGATTACATCTTGTACATGTTTGTTTTATTTTCAGAATCCATTCCTTCCACCATGCATGTTTCAACGTAA
GATTTGTTATTGATAGTATTAATTTTTTTTTTTGTTGACGGATAGTATTGATTTTTAATTACGTTTATTTTGTATTAAC
TTTAAAATAAAAGAACAAAGCCAGCTTAATTTTTAACACTTTCGTATTTGGATAAAAACACAAAACAAAACATTTCGGGT
CCTTTACTTCTGTTTGAGATCAACGTAATGCAACATTTAAAATTTGTAATTGGGAATTCAAACCTAGTCTAAGTTTAAAA
TTAATTTTGAATGACTTAACTGTACAATTCAATACAATACAATGCGCGACAAAATGGAAGACACGTATCAAAGAAATTCC
ACACCCGTTATTGCAGACATTTTTGTTATAATAATACAGGTTTGCTATCTACACATTAATGACGCACAACTATTTAGTTT
CTTAAAAAGACCAAGTGGCAATGTCTATCTACATAAATTGTTTATTCAAAAAATCAATCGCCGAAAATAATAATCTTTAC
AAATTTGCATTTGCCAAAACCTAAGTACTAATACCACGTTGAATGTCATGCAGAACCGAAGAACATGGCGGGGTGAGTGT
ATCTTTAGAGAAATCTGATATAAAATTACGTTTAAAACCAAAATAATTTTATCAATCAGGATTTTTAATTTTAAGTTCAT
TTTATTTTTATTCCATAGATTTATAATTTTCAATCGTAAAAAATAAGTTGAATGCTTAATTAACAACACTGGACACAAGG
GCGCGTCAGCACCTTCAGTTGATGCACTACCCAATCAGATCCCAATGTTGTTTGTTAACAACTGTTAGAGTTGGAACTTT
CCCACTCAGGGCATCAGAATCATCATCACCCTTAACTTAGGGCCCCCCTTTGACATTTTCACAATGTCAATGAAAAAGCC
AAGAGAGAAAGAGAGGGAGAGCATATCTTTCTAACTCCTCCCACCCCTACAAAATTCCACTTTCACATATCTCTTTCTTT

Hap3

TTTTCTCTTTTCAATTTTTCTAGTATAAAATAATTAATTGCCCAACTCCCTCAGTGCTCCACCAACCCCTCTTGCTTCTT
CCTCTTATCCTTCTCTCTAGATTTTGCCATTCACCCTAAAAGTCAGCGCCATCTTTTGTGCCTTATTTCCTCCCTCAGTT
CCGAAACTTTGTTGCTTTAATTGGGAAAAGAAAAGAAAAGAAAAACCACACTGATTTTTATTTTTTTATCTTCCTTAATT
CATAGCCTTACCCAGAAGTCTCTTTCTGCTTTTCTCTCCCTCTGCATA
ATGCATACTAGAACTAAACTTTCTAAAACTCCCAAATTCATATAACTAAGGGTGATCAAATTGGGTCGTTGTTTATCACG
CATTCACAGAAAGTATGATTTTTTTATTTAAATGTTTGCTATAAGAAAATATATCGTTTGCATATAATTTAGACTGCTTC
CTATTTTGAAAGTGTAAAGTCGATAAACGTTTTATAAGTGTTTTATATGTAGGAAGAGTGCAAAGTTTGAGCATGTGAAA
TGCTACAAATTGTGCAAAATTGGCAATGGTGATCATATTTTATTTACACAACATTTTTCAATCTCAACTACTATTGTTCT
TCCATTAGTTTCACTTCCATGCTATTTCTTAATGTCAATCTCAAATTAATTTTTTTTGTCACATATACAAGTATCAAATT
AAGTATAACAACATATAAATAAAAAAGATTCACAAACCTAATGTCTTGAGATTTTTTATTAAAGCATGATGTCAAATTTA
GTTATATAATATTTTTGTGTCCTATTATTAATGAAATCTCCTAGAATCTTATTCAAGTATTTGATGAGGAGTTCTAGAAG
ACAAACTCATTGACAACTTCAACTAGATTTATTATTAAAATATATTTGTATATAAAAATAAAAAAAATTCCCTATTTATA
TAATGCTTTTGTATTTCTCATGAATTTAACTTTTAGAGATAAATAAACCAAATTTGCAATATTTTTAATATTTCTATTGT
TTCAAAATTAAAACATGTATGAATTGCAATTTCAATTTAAAATAAAATAAAAATATAAAAAAAGTTGGATTCACTCTTAG
ATCCACTAAAAAAAGTTACTAATCCAAGTTAATTTCTACCATTAAAGTAAAAATTACAGGTGTTCTTAAATATTACAATA
TATGATAACAAAAGACCCACTAAAAAAAATTAAGAGCTTAAATTAAGTTCTTGCTTTCGAGATAATATGTGTTTGTATTT
ATGGTAGAACTACCATAGCATGAAATCCCATATTATTTCACCACATAAGTATCAAAGTTGATACTTTTAAAAACGTAAAT
TGGATTGTTGTTATTGGCAATCTAAACATACACATACTCAATTTTGAAATGTTATTTTTGTTGTAATTACTGTTATTAGT
TTTTAGTTAGGTTTCGAATAGAACCTTAGAATATCTTCACAAACTTTTTATCATTTGCTTTGGGCTAGTTATAATAATCA
TACTTAAAATTTTTTCGTTGCCTTCTCTCTCAAAATGTAATCCACATAAATTGAATATAGATTTTTTTTCCCCCGCAAAA
TACCCACCCATATCCTTGAAATATTATTTGAGTGCGACTTCGACCATCTTAAAATATTTCAATTTGTTGTTAAACTTCGC
ATCTATGCATTATTGTTTAATTGTGATTTTCTTTTGTCACATTCCACATATTAGAATTCAAATTTAAATCCACCTCTGCA
GTTCTACTTCTTGCTTTGTTCAACTCCAACGGTCCTACCACATACCATTATTATTAGTAGTAGTAATAATAGTTTAACTT
ACATACATTATTAATGTAAAACATATTTATACATACACTCGTTATTAATTAATTTCCACCTATGCATTGATATAAAGTGA
GGATAAAGTAAATTTGTATTACATATCCATGTAAGTTTAATTTATAATAACTATATAATTATATATAAATTAAATTCTAT
TATATTGATTACATCTTGTACATGTTTGTTTTATTTTCAGAATCCATTCCTTCCACCATGCATGTTTCAACGTAAGATTT

Hap4	GTTATTGATAGTATTAATTTTTTTTTTTTGTTGACGGGATAGTATTGATTTTTAATTACGTTTATTTTGTATTAACTTTA<br>AAATAAAAGAACAAAGCCAGCTTAATTTTTAACACTTTCGTATTTGGATAAAAACACAAAACAAAACATTTCGGGTCCTT<br>TACTTCTGTTTGAGATCAACGTAATGCAACATTTAAAATTTGTAATTGGGAATTCAAACCTAGTCTAAGTTTAAAATTAA<br>TTTTGAATGACTTAACTGTACAATTCAATACAATACAATGCGCGACAAAATGGAAGACACGTATCAAAGAAATTCCACAC<br>CCGTTATTGCAGACATTTTTGTTATAATAATACAGGTTTGCTATCTACACATTAATGACGCACAACTATTTAGTTTCTTA<br>AAAAGACCAAGTGGCAATGTCTATCTACAAAAATTGTTTATTCAAAAAATCAATCGCCGAAAATAATAATCTTTACAAAT<br>TTGCATTTGCCAAAACCTAAGTACTAATTCCACGTTGAATGTCATGCAGAACCGAAGAACATGGCGGGGTGAGTGTATCT<br>TTAGAGAAATCTGATATAAAATTACGTTTAAAACCAAAATAATTTTATCAATCAGGATTTTTAATTTTAAGTTCATTTTA<br>TTTTTATTCCATAGATTTATAATTTTCAATCGTAAAAAATAAGTTGAATGCTTAATTAACAACACTGGACACAAGGGCGC<br>GTCAGCACCTTCAGTTGATGCACTACCCAATCAGATCCCAATGTTGTTTGTTAACAACTGTTAGAGTTGGAACTTTCCCA<br>CTCAGGGCATCAGAATCATCATCACCCTTAACTTAGGGCCCCCCTTTGACATTTTCACAATGTCAATGAAAAAGCCAAGA<br>GAGAAAGAGAGGGAGAGCATATCTTTCTAACTCCTCCGACCCCTACAAAATTCCACTTTCACATATCTCTTTCTTTTTTT<br>CTCTTTTCAATTTTTCTAGTATAAAATAATTAATTGCCCAACTCCCTCAGTGCTCCACCAACCCCTCTTGCTTCTTCCTC<br>TTATCTTTCTCTCTAGATTTTGCCATTCACCCTAAAAGTCAGCGCCATCTTTTGTGCCTTATTTCCTCCCTCAGTTCCGA<br>AACTTTGTTGCTTTAATTGGGAAAAGAAAAGAAAAGAAAAACCACACTGATTTTTATTTTTTTATCTTCCTTAATTCATA<br>GCCTTACCCAGAAGTCTCTTTCTGCTTTTCTCTCCCTCTGCATA<br>ATGCATACTAGAACTAAACTTTCTAAAACTCCCAAATTCATATAACTAAGGGTGATCAAATTGGGTCGTTGTTTATCACG<br>CATTCACAGAAAGTATGATTTTTTTATTTAAATGTTTGCTATAAGAAAATATATCGTTTGCATATAATTTAGACTGCTTC<br>CTATTTTGAAAGTGTAAAGTCGATAAACGTTTTATAAGTGTTTTATATGTAGGAAGAGTGCAAAGTTTGAGCATGTGAAA<br>TGCTACAAATTGTGCAAAATTGGCAATGGTGATCATATTTTATTTACACAACATTTTTCAATCTCAACTACTATTGTTCT<br>TCCATTAGTTTCACTTCCATGCTATTTCTTAATGTCAATCTCAAATTAATTTTTTTTGTCACATATACAAGTATCAAATT<br>AAGTATAACAACATATAAATAAAAAAGATTCACAAACCTAATGTCTTGAGATTTTTTATTAAAGCATGATGTCAAATTTA<br>GTTATATAATATTTTTGTGTCCTATTATTAATGAAATCTCCTAGAATCTTATTCAAGTATTTGATGAGGAGTTCTAGAAG<br>ACAAACTCATTGACAACTTCAACTAGATTTATTATTAAAATATATTTGTATATAAAAATAAAAAAAATTCCCTATTTATA<br>TAATGCTTTTGTATTTCTCATGAATTTAACTTTTAGAGATAAATAAACCAAATTTGCAATATTTTTAATATTTCTATTGT<br>TTCAAAATTAAAACATGTATGAATTACAATTTCAATTTAAAATAAAATAAAAATATAAAAAAAGTTGGATTCACTCTTAG

ATCCACTAAAAAAAGTTACTAATCCAAGTTAATTTCTACCATTAAAGTAAAAATTACAGGTGTTCTTAAATATTACAATA
TATGATAACAAAAGACCCACTAAAAAAAATTAAGAGCTTAAATTAAGTTCTTGCTTTCGAGATAATATGTGTTTGTATTT
ATGGTAGAACTACCATAGCATGAAATCCCATATTATTTCACCACATAAGTATCAAAGTTGATACTTTTAAAAACGTAAAT
TGGATTGTTGTTATTGGCAATCTAAACATACACATACTCAATTTTGAAATGTTATTTTTGTTGTAATTACTGTTATTAGT
TTTTAGTTAGGTTTCGAATAGAACCTTAGAATATCTTCACAAACTTTTTATCATTTGCTTTGGGCTAGTTATAATAATCA
TACTTAAAATTTTTTCGTTGCCTTCTCTCTCAAAATGTAATCCACATAAATTGAATATAGATTTTTTTCCCCCGCAAAA
TACCCACCCATATCCTTGAAATATTATTTGAGTGCGACTTCGACCATCTTAAAATATTTCAATTTGTTGTTAAACTTCGC
ATCTATGCATTATTGTTTAATTGTGATTTTCTTTTGTCACATTCCACATATTAGAATTCAAATTTAAATCCACCTCTGCA
GTTCTACTTCTTGCTTTGTTCAACTCCAACGGTCCTACCACATACCATTATTATTAGTAGTAGTAATAATAGTTTAACTT
ACATACATTATTAATGTAAAACATATTTATACATACACTCGTTATTAATTAATTTCCACCTATGCATTGATATAAAGTGA
GGATAAAGTAAATTTGTATTACATATCCATGTAAGTTTAATTTATAATAACTATATAATTATATATAAATTAAATTCTAT
TATATTGATTACATCTTGTACATGTTTGTTTTATTTTCAGAATCCATTCCTTCCACCATGCATGTTTCAACGTAAGATTT
GTTATTGATAGTATTAATTTTTTTTTTTTTGTTGACGGGATAGTATTGATTTTTAATTACGTTTATTTTGTATTAACTTTA
AAATAAAAGAACAAAGCCAGCTTAATTTTTAACACTTTCGTATTTGGATAAAAACACAAAACAAAACATTTCGGGTCCTT
TACTTCTGTTTGAGATCAACGTAATGCAACATTTAAAATTTGTAATTGGGAATTCAAACCTAGTCTAAGTTTAAAATTAA
TTTTGAATGACTTAACTGTACAATTCAATACAATACAATGCGCGACAAAATGGAAGACACGTATCAAAGAAATTCCACAC
CCGTTATTGCAGACATTTTTGTTATAATAATACAGGTTTGCTATCTACACATTAATGACGCACAACTATTTAGTTTCTTA
AAAAGACCAAGTGGCAATGTCTATCTACATAAATTGTTTATTCAAAAAATCAATCGCCGAAAATAATAATCTTTACAAAT
TTGCATTTGCCAAAACCTAAGTACTAATTCCACGTTGAATGTCATGCAGAACCGAAGAACATGGCGGGGTGAGTGTATCT
TTAGAGAAATCTGATATAAAATTACGTTTAAAACCAAAATAATTTTATCAATCAGGATTTTTAATTTTAAGTTCATTTTA
TTTTTATTCCATAGATTTATAATTTTCAATCGTAAAAAATAAGTTGAATGCTTAATTAACAACACTGGACACAAGGGCGC
GTCAGCACCTTCAGTTGATGCACTACCCAATCAGATCCCAATGTTGTTTGTTAACAACTGTTAGAGTTGGAACTTTCCCA
CTCAGGGCATCAGAATCATCATCACCCTTAACTTAGGGCCCCCCTTTGACATTTTCACAATGTCAATGAAAAAGCCAAGA
GAGAAAGAGAGGGAGAGCATATCTTTCTAACTCCTCCGACCCCTACAAAATTCCACTTTCACATATCTCTTTCTTTTTTT
CTCTTTTCAATTTTTCTAGTATAAAATAATTAATTGCCCAACTCCCTCAGTGCTCCACCAACCCCTCTTGCTTCTTCCTC
TTATCTTTCTCTCTAGATTTTGCCATTCACCCTAAAAGTCAGCGCCATCTTTTGTGCCTTATTTCCTCCCTCAGTTCCGA

AACTTTGTTGCTTTAATTGGGAAAAGAAAAGAAAAACCACACTGATTTTTATTTTTTTATCTTCCTTAATTCATAGCCTT
ACCCAGAAGTCTCTTTCTGCTTTTCTCTCCCTCTGCATA

Table S5. Promoter elements analysis of different haplotypes

No.	Hap1	Hap2	Hap3	Hap4
1	+ AAGAA-motif	+ AAGAA-motif	+ AAGAA-motif	+ AAGAA-motif
2	+ ABRE	+ ABRE	+ ABRE	+ ABRE
3	+ ABRE3a	+ ABRE3a	+ ABRE3a	+ ABRE3a
4	+ ABRE4	+ ABRE4	+ ABRE4	+ ABRE4
5	+ AC-II	+ AC-II	+ AC-II	+ AC-II
6	+ ACE	+ ACE	+ ACE	+ ACE
7	+ ARE	+ ARE	+ ARE	+ ARE
8	+ ATCT-motif	+ ATCT-motif	+ ATCT-motif	+ ATCT-motif
9	+ AT~ABRE	+ AT~ABRE	+ AT~ABRE	+ AT~ABRE
10	+ AT~TATA-box	+ AT~TATA-box	+ AT~TATA-box	+ AT~TATA-box
11	+ Box 4	+ Box 4	+ Box 4	+ Box 4
12	+ CAAT-box	+ CAAT-box	+ CAAT-box	+ CAAT-box
13	+ CARE	+ CARE	+ CARE	+ CARE
14	+ CAT-box	+ CAT-box	+ CAT-box	+ CAT-box
15	+ CCAAT-box	+ CCAAT-box	+ CCAAT-box	+ CCAAT-box
16	+ CGTCA-motif	+ CGTCA-motif	+ CGTCA-motif	+ CGTCA-motif
17	+ ERE	+ ERE	+ ERE	+ ERE
18	+ G-Box	+ G-Box	+ G-Box	+ G-Box
19	+ G-box	+ G-box	+ G-box	+ G-box
20	+ GATA-motif	+ GATA-motif	+ GATA-motif	+ GATA-motif

21	+	GC-motif		no	+	GC-motif		no
22	+	LTR	+	LTR	+	LTR	+	LTR
23	+	MBS	+	MBS	+	MBS	+	MBS
24	+	MRE	+	MRE	+	MRE	+	MRE
25	+	MSA-like	+	MSA-like	+	MSA-like	+	MSA-like
26	+	MYB recognition site	+	MYB recognition site	+	MYB recognition site	+	MYB recognition site
27	+	MYC	+	MYC	+	MYC	+	MYC
28	+	Myb	+	Myb	+	Myb	+	Myb
29	+	O2-site	+	O2-site	+	O2-site	+	O2-site
30	+	RY-element	+	RY-element	+	RY-element	+	RY-element
31	+	SARE	+	SARE	+	SARE	+	SARE
32	+	STRE	+	STRE	+	STRE	+	STRE
33	+	TATA	+	TATA	+	TATA	+	TATA
34	+	TATA-box	+	TATA-box	+	TATA-box	+	TATA-box
35	+	TCA-element	+	TCA-element	+	TCA-element	+	TCA-element
36	+	TCCC-motif	+	TCCC-motif	+	TCCC-motif	+	TCCC-motif
37	+	TCT-motif	+	TCT-motif	+	TCT-motif	+	TCT-motif
38	+	TGA-element	+	TGA-element	+	TGA-element	+	TGA-element
39	+	TGACG-motif	+	TGACG-motif	+	TGACG-motif	+	TGACG-motif
40	+	Unnamed__1	+	Unnamed__1	+	Unnamed__1	+	Unnamed__1
41	+	Unnamed__10			+	Unnamed__10		
42	+	Unnamed__12			+	Unnamed__12		
43	+	Unnamed__14			+	Unnamed__14		
	+	Unnamed__4	+	Unnamed__4	+	Unnamed__4	+	Unnamed__4
	+	Unnamed__6	+	Unnamed__6	+	Unnamed__6	+	Unnamed__6

+ Unnamed__8		+ Unnamed__8	
+ WRE3	+ WRE3	+ WRE3	+ WRE3
+ as-1	+ as-1	+ as-1	+ as-1
