# Supplementary figures and images for: Identifications of QTLs and Candidate Genes Associated with Pseudomonas syringae Responses in Cultivated Soybean (Glycine max) and Wild Soybean (Glycine soja)
Source: Int J Mol Sci. 2023 Feb 27;24(5):4618. doi: 10.3390/ijms24054618 (PMC10003559; doi:10.3390/ijms24054618)

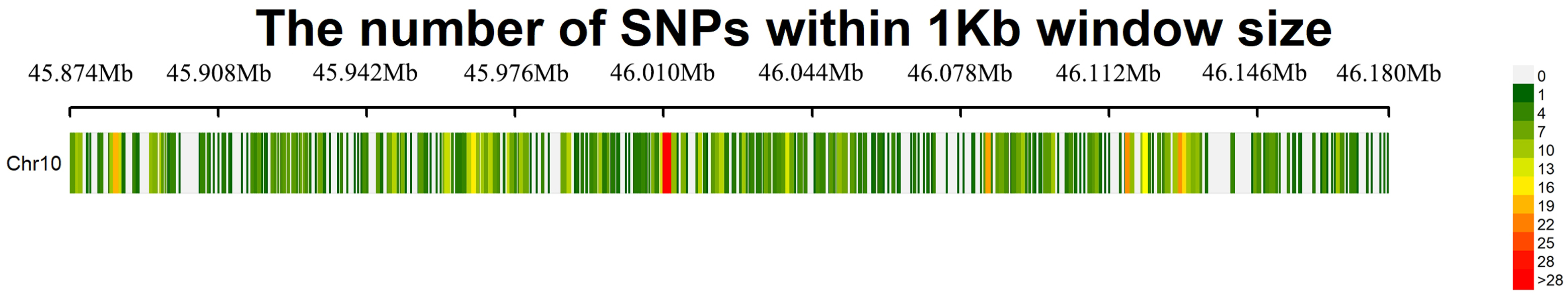

Supplement: Supplementary file 1 [file ijms-24-04618-s001.zip › Figure S1.jpg]
